# A Comprehensive Study on the Dye Adsorption Behavior of Polyoxometalate-Complex Nano-Hybrids Containing Classic β-Octamolybdate and Biimidazole Units

**DOI:** 10.3390/molecules24040806

**Published:** 2019-02-22

**Authors:** Shuang Liang, Yan-Mei Nie, Sang-Hao Li, Jian-Liang Zhou, Jun Yan

**Affiliations:** 1 School of Chemistry and Chemical Engineering, Central South University, Changsha 410083, China; liang324@csu.edu.cn (S.L.); 15367493565@163.com (Y.-M.N.); 172311031@csu.edu.cn (S.-H.L.); zhoujl@csu.edu.cn (J.-L.Z.); 2 Hunan Provincial Key Laboratory of Efficient and Clean Utilization of Manganese Resources, Central South University, Changsha 410083, Hunan, China

**Keywords:** polyoxometalate, octamolybdate, dye adsorption

## Abstract

Six new hybrids based on β-[Mo_8_O_26_]^4−^ polyoxometalates, [Ni(H_2_biim)_3_]_2_[β-Mo_8_O_26_]•8DMF(**1**); (DMA)_2_[M(H_2_biim)_2_(H_2_O)_2_][β-Mo_8_O_26_]•4DMF (M = Ni (**2**), Co (**3**)), DMA = dimethyl-ammonium, H_2_biim=2,2′-biimidazole); [M(H_2_biim)(DMF)_3_]_2_[β-Mo_8_O_26_]•2DMF (M = Zn (**4**), Cu (**5**)); [(DMA)_2_[Cu(DMF)_4_][β-Mo_8_O_26_]•2DMF]_n_ (**6**), have been successfully synthesized and characterized. Compounds **2–5** show favorable capacity to adsorb methylene blue (MB) at room temperature, and they can selectively capture MB molecules from binary-mixture solutions of MB/MO (MO = Methyl Orange), or MB/RhB (RhB = Rhodamine B). Compound **3** can uptake up to 521.7 mg g^−1^ MB cationic dyes rapidly, which perform the maximum adsorption in an hour among the reported materials as far as we know. The compounds are stable and still work very efficiently after three cycles. For compound **3**, the preliminary adsorption mechanism studies indicated that the adsorption is an ion exchange process and the adsorption behavior of polyoxometalate-complex can be benefited from additional exchangeable protons in the complex cations.

## 1. Introduction

Nowadays, the removal of organic dyes from wastewater is an ongoing hot topic in many fields, due to their high toxicity and potential carcinogenic effects [1,2,3,4,5,6,7]. Several techniques are available for the treatment of the dyes such as an electrochemical technique destroying the color groups [8], a bio-degradation process mineralizing the colorless organic intermediates [9], chemical oxidation [10,11], photocatalytic degradation [12,13,14], membrane filtration [15], liquid–liquid extraction [16], and adsorption [17,18,19]. Among these methods, solid-liquid adsorption technology is regarded as one with the most traditional and competitive methods [20,21], because it is easy and safe to perform, has a high efficiency, simplicity of design, ease of operation and resilience to toxic substances [22], and is comparatively eco-friendly. Various adsorbents, such as activated carbon [23], nanoporous silica [24], clay materials, solid waste [25], MOFs [26,27], and different types of biomaterials [28,29,30,31] have been used to remove dyes from sewage. However, most of these adsorbents suffer from low adsorption capacities [32,33], poor selectively or inconvenient separation and recycle procedures and some adsorbents might cause secondary environmental contamination if they are not fully recovered from the environment. The development of stable dye sorbents that have high adsorption capacities, good removal efficiency, and can be easily separated is much anticipated, both in scientific and in industrial applications [34].

In contrast, polyoxometalate (POM)-based sorbent materials with hierarchical structures have evoked considerable interest owing to their outstanding features such as: (1) large surface area, (2) controllable shape and size with good stability, (3) large expandable intermolecular free space, (4) oxo-enriched surfaces with highly electronegative bonding sites and (5) high separation efficiency with improved reusability [35,36,37,38,39,40,41,42,43,44,45,46,47,48,49]. Therefore, POMs, especially POM-based organic–inorganic hybrid materials, have been considered some of the most promising sorbents for the selective separation of cationic dyes [50,51,52,53,54,55,56,57,58,59,60,61,62,63,64]. Su’s group has reported a series of new POMs and POM hybrid with rapids and large-scale adsorption properties [65]. Wang’s group also recently reported a POM@MOF hybrid sorbent, and various POMs incorporated into MIL-101 can improve the dye adsorption capacity [66,67]. However, the adsorption/desorption mechanism in this system is still not well understood and normally simply described as an ion-exchange process.

Towards developing POM-based hybrid absorbent materials with improved capacities and removal efficiency and understanding the adsorption process, we intend to simplify the model and use the classic β-octamolybdate as basic POM building unit to further explore the correlation between the adsorption and hybrid structures. The functional cations were used transition metal complexes about 2,2′-biimidazole ligand (H_2_biim). As an organic ligand, imidazole is a good electron donor for sp*^2^* hybrid N atom in unsaturated system. It is able to coordinate to transition metals with three reversible types of binding: neutral (H_2_biim), monoanion (Hbiim^−^), and dianion (biim^2−^) types, but few compounds involving transition metals, H_2_biim ligands, and polyoxoanions have been reported [68,69,70]. The N–H bond of the ligand can form supramolecular compounds [71], which is beneficial to the ion-exchange and adsorption process of cationic dye in a wastewater. Herein, by adjusting the synthetic parameters and transition metal centers, six new polyoxometalate-complex nano-hybrids with different number of H_2_biim were isolated and characterized, including [Ni(H_2_biim)_3_]_2_[β-Mo_8_O_26_]•8DMF (**1**); (DMA)_2_[M(H_2_biim)_2_(H_2_O)_2_][β-Mo_8_O_26_]•4DMF (M = Ni (**2**), Co (**3**)) (DMA = dimethyl-ammonium); [M(H_2_biim)(DMF)_3_]_2_[β-Mo_8_O_26_]•2DMF (M = Zn (**4**), Cu (**5**)); [(DMA)_2_[Cu(DMF)_4_][β-Mo_8_O_26_]•2DMF]_n_ (**6**) Complexes 2–5 show rapid and large-scale adsorption behaviors and good selective separation of cationic dyes. Meanwhile, the detailed complex structure indeed affects the adsorption efficiency and stability as expected. Particularly, compound **3** can uptake up to 521.7 mg g^−1^ MB^+^ cationic dye from a wastewater with excellent adsorbent efficiency, and performs the best among all the reported materials as far as we know. The primarily adsorption mechanism study indicated that the process is an ion-exchange process, and the adsorbed dyes can be easily desorbed. The compounds are quite stable and still work very efficiently after three cycles.

## 2. Results and Discussion

### 2.1. Synthetic Strategy

**Scheme 1:**
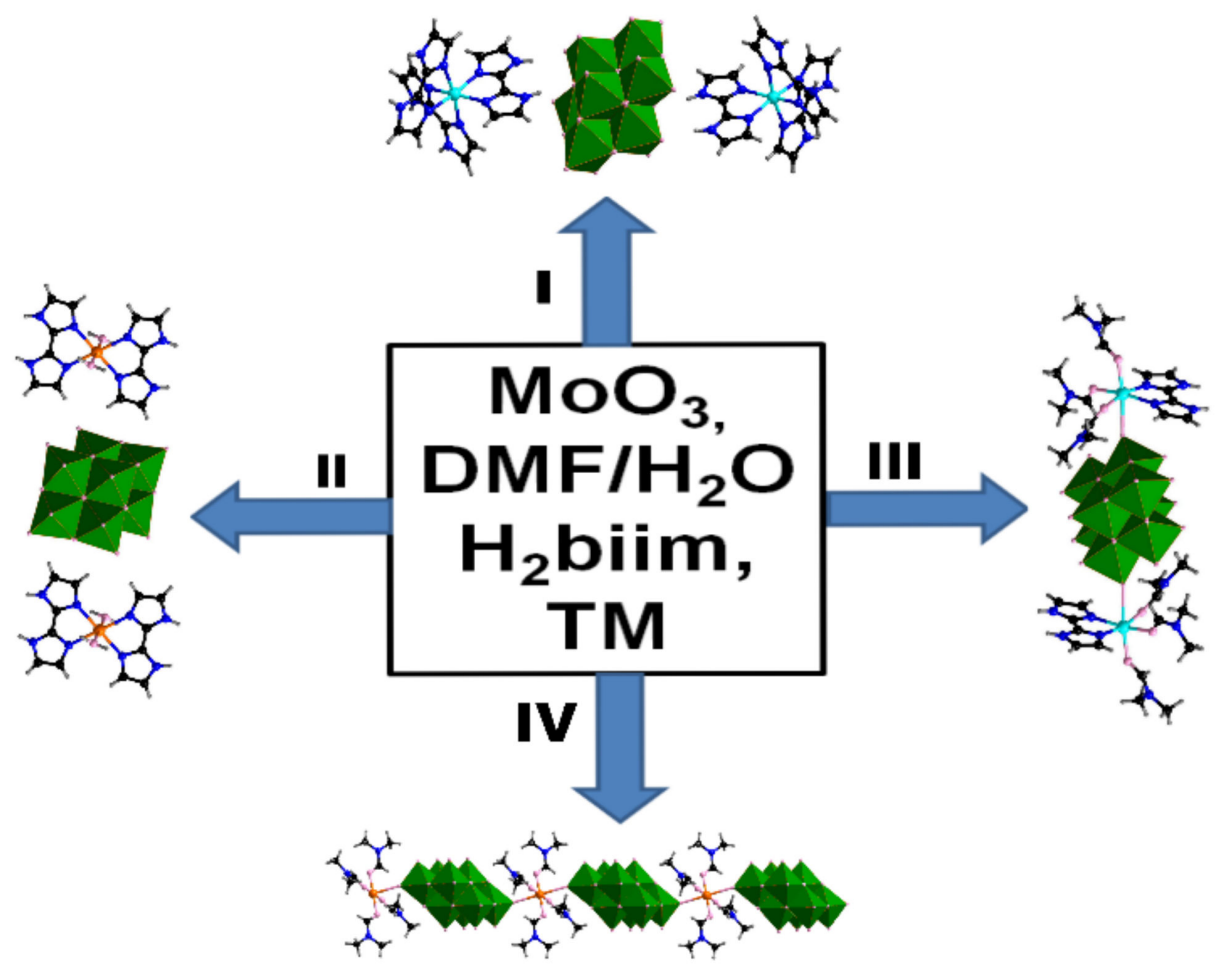
The four different types of POM-complex hybrid molecules were obtained in the studied system. Type I: TM = Ni, V_DMF_/V_H2O_ = 4/1, M_TM_/M_H2biim_=1/3; Type II: TM = Ni or Co, V_DMF_/V_H2O_ = 7/3, M_Co_/M_H2biim_ = 16/3 for compound 3, M_Ni_/M_H2biim_ = 8/3 for compound 2; Type III: TM = Cu or Zn, V_DMF_/V_H2O_ = 7/3 and M_Zn_/M_H2biim_ = 3/1 for compound 4; V_DMF_/V_H2O_ = 4/1 and M_Cu_/M_H2biim_ = 10/3 for compound 5; Type **IV**: TM = Cu,V_DMF_/V_H2O_ = 4/1 and M_Cu_/M_H2biim_ = 10/3.The β-[Mo_8_O_26_]^4−^ was shown in polyhedron mode and the complex cations were shown ball and stick mode.

Accurate control of the synthesis of POM-complex hybrids is not always possible because many subtle factors of the experimental parameter may affect their assembly. In this work, complexes **1**–**6** were obtained under similar experimental conditions with different metal salts, resulting in noticeably different structures. MoO_3_ was used as Mo source instead of Na_2_MoO_4_ or (NH_4_)_6_[Mo_7_O_24_] to avoid an additional cation that could affect the self-assembly process and change the solubility of the complex hybrid. For dye sorbent material design, high temperature reactions in DMF/H_2_O mixture were used to form complexes with fewer coordinated water molecules in the final crystal structure. A lattice DMF with bigger size and weaker H-bond to POM unit may also benefit the adsorption/desorption process. The DMA cation comes from the thermal decomposition of DMF [72,73]. Further, the H_2_biim ligand can not only stabilize the transition metal center of the complex which further tune the solubility of the crystals, but also plays the role of proton donor and morphology-control-factor of the supramolecular structures. Additionally, although the H_2_biim ligands adopt unified bidentate ligand mode in compounds **1–5** and the POM units are strictly the same β-[Mo_8_O_26_]^4−^, parallel experiments indicated that the metal ions were crucial for the formation of the title complexes, as shown in Scheme 1. When Ni^II^ ion was used as central metal, the simple [Ni(H_2_biim)_3_]^2+^ cation was formed (compound **1**). As the concentration of Ni^II^ and ratio of H_2_O/DMF increased, the cation forms [Ni(H_2_biim)_2_(H_2_O)_2_]^2+^ (compound **2**) with two organic ligands and two water molecules. By replacing the Ni^II^ with Co^II^ and changing the ratio of solvent to ligand, the Co^II^ version analogue of **2** can be isolated as compound **3**. Also, when Zn^II^ ions or Cu^II^ was used instead of NI^II^ ion, the metal ion coordinated with a single H_2_biim ligand, three DMF molecules and the β-[Mo_8_O_26_]^4−^ in compounds **4** and **5**. In the case of compound **6**, the Cu^II^ coordinated with four DMF molecules and two β-[Mo_8_O_26_]^4−^ units, and the compound was isolated as a by-product with compound **5**. Clearly, it is believed that H_2_O, DMF and H_2_biim are competitive ligands in this synthetic system, and the fine-tuning structure of the POM-complex hybrid could be realized by simply adjusting the concentration of reactant and the reaction solvent.

### 2.2. Description of Crystal Structures of Complexes

The single crystal structure data reveals that compound **1** crystallizes in the monoclinic system with space group *P_21/c_*. The asymmetric unit of compound **1** contains half of the β-[Mo_8_O_26_]^4−^ anion, one Ni^II^ ion adopts a six coordinated octahedron structure by three poly-nitrogen heterocyclic H_2_biim ligands with Ni–N bond with distances varying from 2.076(1)–2.121(1) Å (Supplementary Figure S3), and four lattice DMF molecules.

**Figure 1:**
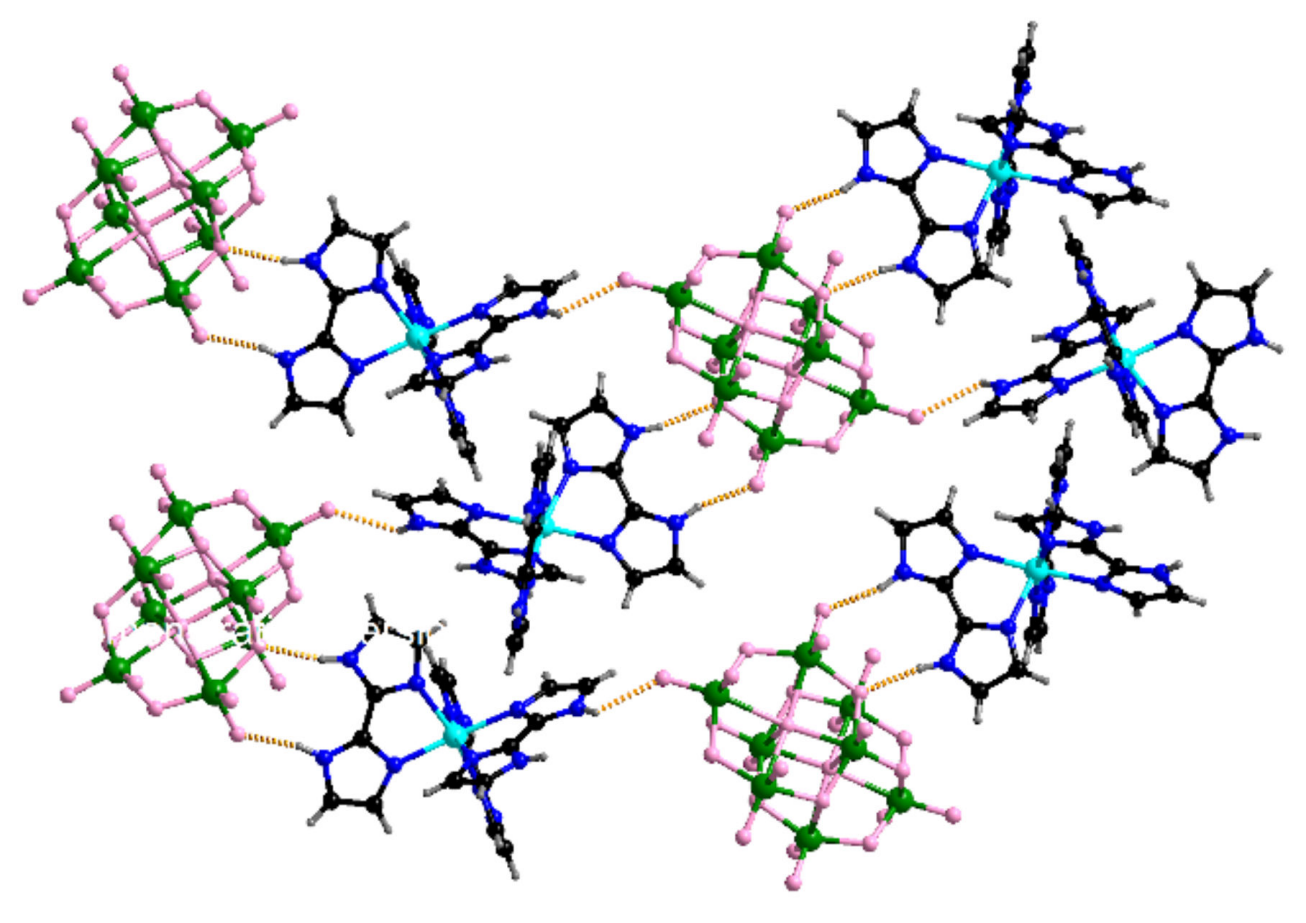
The structural view of H-bond between POM and [Ni(H_2_biim)_3_]^2+^ cation in compound **1**. The H-bond was shown in dashed line (colors code: Mo—green; Ni—cyan; O–pink; N–blue; C–black; H–grey).

As depicted in Figure 1 and Supplementary Figure S1, there are two types of strong H-bonds between POM anion and nearby complex cations with average N–H^…^O distance of 2.85(1) Å. Each [Ni(H_2_biim)_3_]^2+^ cation forms three H-bond with two β-[Mo_8_O_26_]^4−^ next to it and therefore they constructed a 2D supramolecular network. The lattice DMF molecules lay between the layers. The bond lengths around the Ni^II^ atoms are listed in Supplementary Table S3.

**Figure 2:**
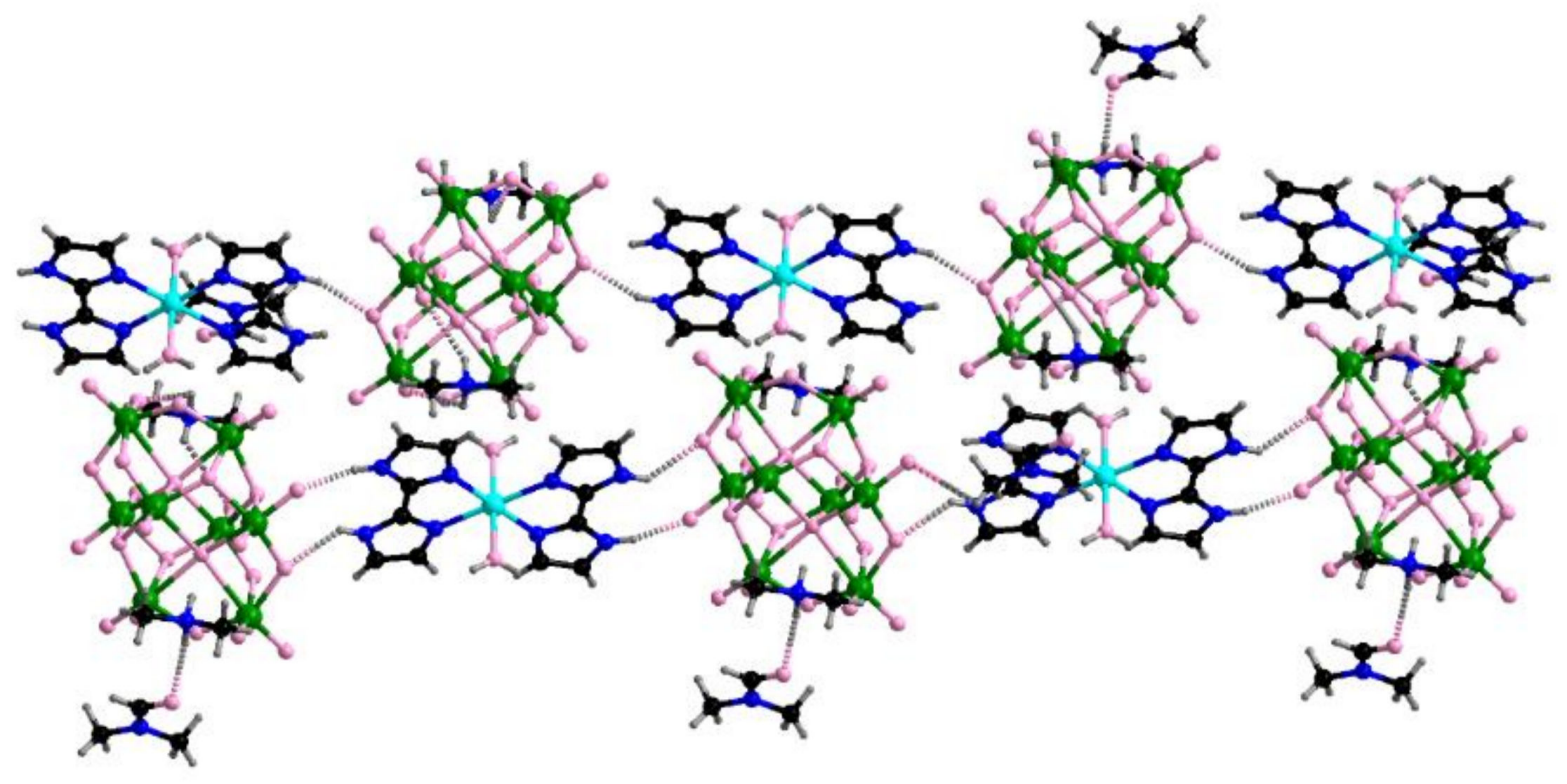
The structural view of the 1D supramolecular chain of POM and [Ni(H_2_biim)_2_(H_2_O)_2_]^2+^ cation in compound **2**. The H-bond was shown in dashed line (Colors code: Mo—green; Ni—cyan; O—pink; N—blue; C—black; H—grey).

Alternatively, two H_2_biim ligands and two water molecules coordinated Ni^II^ in *trans*- mode and formed [Ni(H_2_biim)_2_(H_2_O)_2_]^2+^ cationic complex in compound **2**. The Ni^II^ is six-coordinated with two molecule of H_2_O through Ni–O (2.062(1), 2.082(1) Å) and two molecules through Ni–N bond varying from 2.078(2) to 2.125(1) Å. As shown in Figure 2 and Supplementary Figure S2, there are also two kinds of H-bonding mode between the complex and POM units. The first type is that two H-bonding links a single H_2_biim ligand and two terminal oxygen form one {MoO_6_} units of POM with average N–H^…^O distance of 2.820(1) Å, which further bond the two parts and form a 1D supramolecular chain. The other mode shows a single H-bond connected to one bridging oxygen ligand of the POM from NH group, which also produce a 1D chain. These two types of anionic chains alternately arrange in the crystal and emerge a 2D layer, and the DMA cations and DMF solvent fill the space between the layers. Compound 3 shows very similar solid structure compared to compound **2**, except the Ni^II^ was replaced by Co^II^.

**Figure 3:**
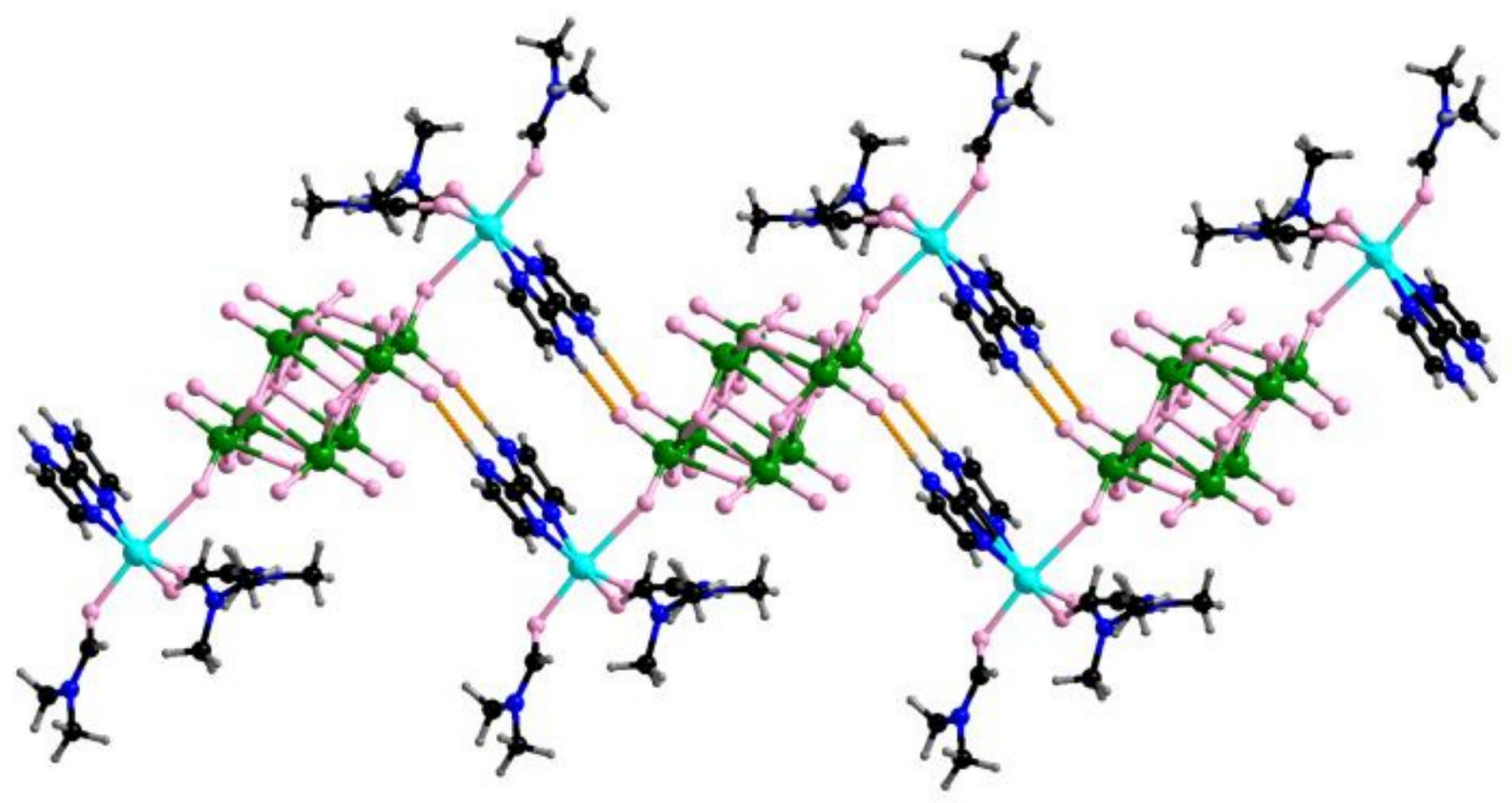
The structural view of H-bonds between neutral [Zn(H_2_biim)(DMF)_3_]_2_[Mo_8_O_26_] molecules in compound **4**. The H-bond was shown in dashed line (Colors code: Mo—green; Zn—light blue; O—pink; N—blue; C—black; H—grey).

When Zn^II^ is used to replace Ni^II^ in **1**, there are less H_2_biim ligands coordinated to transition metal ion cations. As a result, compound **4** containing a neutral [Zn(H_2_biim)(DMF)_3_]_2_[Mo_8_O_26_] hybrid is obtained. Single-crystal X-ray diffraction analysis reveals that the asymmetric unit of complex **4** contains half a β-[Mo_8_O_26_]^4−^ anion, one Zn^II^ ion coordinated with one neutral H_2_biim ligands, three DMF molecules, and a terminal oxygen of the β-[Mo_8_O_26_]^4−^, as well as one lattice DMF molecule. As shown in Figure 3, the Zn^II^ ion is six-coordinate in a distorted octahedral geometry, the bond linking Zn and POM (2.50(1) Å) is much longer than the other five bonds. All of these bond lengths and angles are within the normal ranges observed in other Zn^II^-containing complexes [74]. The H-bonding shows a different mode compared with compounds **1** and **2**. Two parallel H-bonds are formed between the NH piece of coordinated H_2_biim ligand and two terminal oxygen atoms from two {MoO_6_} units of POM cluster, which produce a 1D supramolecular chain, and the lattice DMF molecules arranged orderly in the space between the chains.

**Figure 4:**
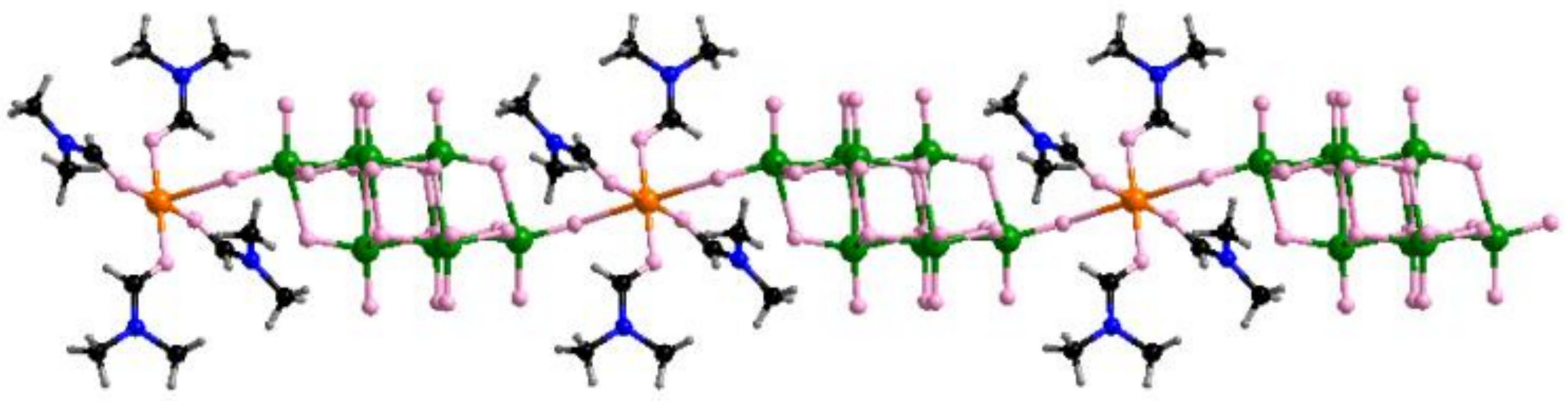
The structural view of 1D coordination chain of {[Cu(DMF)_4_]_n_[Mo_8_O_26_]_n_}^2n−^ in compound **6**. (Colors code: Mo—green; Cu—orange; O—pink; N—blue; C—black; H—grey).

Additionally, when Cu^II^ was used in the synthesis, compounds **5** and **6** were obtained in a single reaction. Compound **5**, [Cu(H_2_biim)(DMF)_3_]_2_[β-Mo_8_O_26_]•2DMF, is an analogue of compound **4**. Compound **6** with the formula {(DMA)_2_[Cu(DMF)_4_][β-Mo_8_O_26_]•2DMF}_n_ is constructed by an anionic 1D coordination polymer {[Cu(DMF)_4_]_n_[Mo_8_O_26_]_n_}^2n−^. As shown in Figure 4, the Cu^II^ ion is six-coordinate in a distorted octahedral geometry with two [Mo_8_O_26_]^4−^ and four DMFs in a *trans*- mode, and the two bonds linking Cu^II^ and POM (2.39(1) Å) is much longer than the other four bonds due to Jahn-Teller effect. No H_2_biim ligands were detected in this structure.

### 2.3. PXRD Patterns and Thermal Analyses

Powder X-ray diffraction (XRD) measurements of compounds **1**–**6** were determined at room temperature (Supplementary Figures S3–S8), and the diffraction peaks positions of the experimental XRD patterns is in agreement with this of the simulated XRD patterns, which indicating the phase purifies of compounds **1**–**4**. Some shifted peaks were also detected in the data due to the moisture absorption in the case of **2**–**5**. In the case of compound **5**, the crystals were separate manually from compound **6**. The XRD shows some more peaks than the simulated patterns, which indicated the exitance of small amounts of compound **6** impurities. The thermal stabilities of the compounds were investigated by thermogravimetric analysis. The TG curves of compound **1** to reveal the weight loss at 100–300 °C of 19.15%, corresponding to the loss of eight DMF molecules (*cal*. 19.76 %) and the compound decomposed at into 600 °C (Supplementary Figure S9). There were three steps of weight loss during the decomposition of compound **2** (Supplementary Figure S10). The TG curve reveals the weight loss at 100–400 °C, a weight loss of 22.45%, corresponding to the loss of four DMF, two DMA and two waters (*cal*. 21.76 %). Although the structure of compound **3** is highly similar to that of **2**, the decomposition process is different (Supplementary Figure S11), the TG curve reveals the weight loss of 21.81 % at 100–300 °C corresponding to the loss of four DMF, two DMA and two waters (*cal*. 21.90%). In the case of compounds **4** and **5** (Supplementary Figures S12 and S13), the first weight loss at 200–500 °C, 26.39% for **4** (*cal*. 26.99%) and 26.94% for **5** (*cal*. 27.03%), which correspond to the loss of free and coordinated DMF. For compound **6**, as shown in Supplementary Figure S14, the weight loss at 100–200 °C of 10.85%, which correspond to the loss of free DMF and DMA (*cal*. 10.20 %). There is a weight loss about 17.01% at 250–550 °C that may correspond to the elimination of coordinated DMF (*cal*. 16.48 %).

### 2.4. Adsorption of Organic Dyes

**Figure 5:**
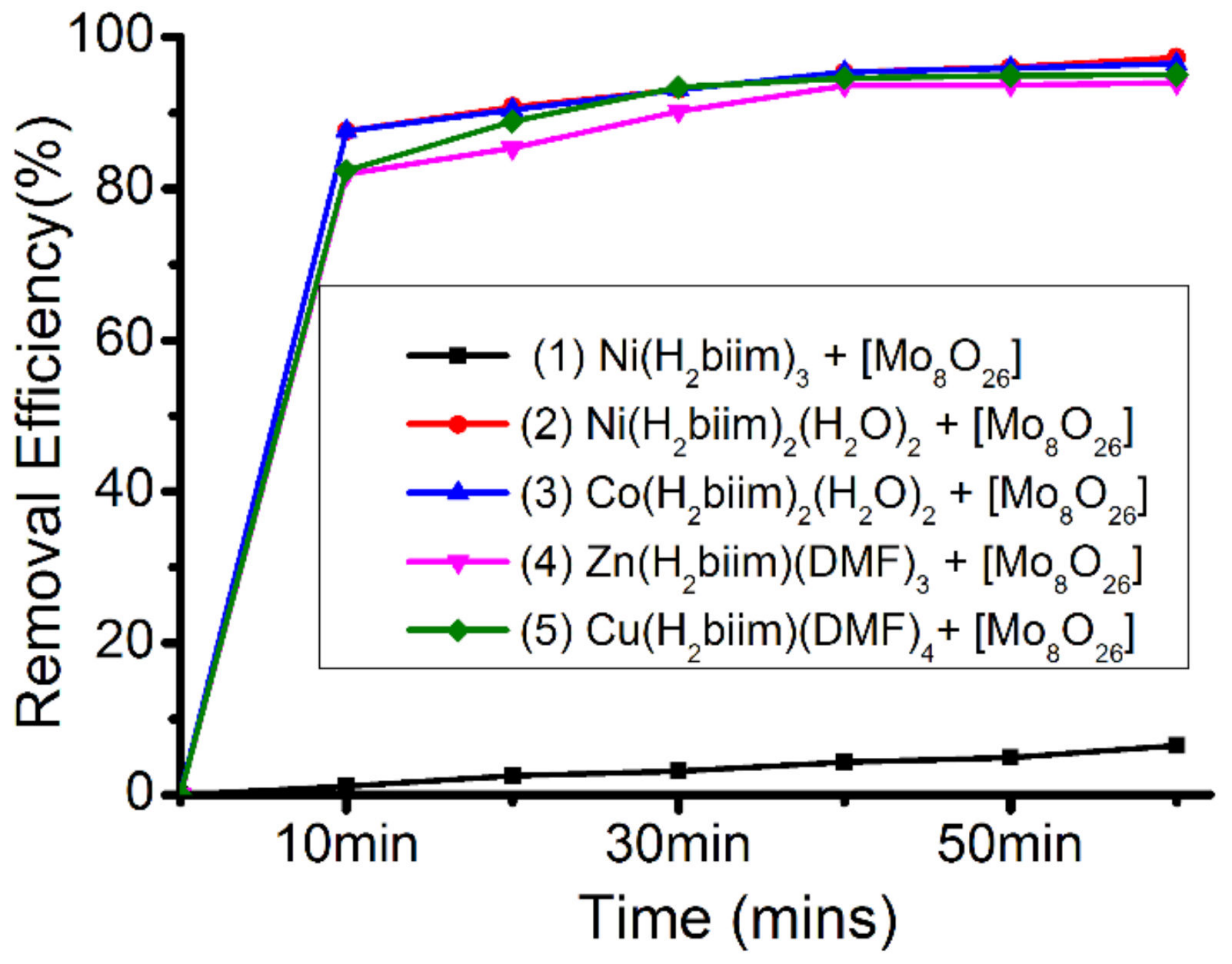
The removal efficiency of compounds **1–**5 in 10 mg L^−1^ methylene blue solution during the first hour.

Recently, potential applications of POM-based composites as organic dye adsorbents have been attracting much attention [75,76]. The removal efficiency and the specific capacity of the adsorbent have been considered the major performance index. Therefore, methylene blue (MB) was selected as a model pollutant to investigate the adsorption behavior of complexes **1**–**5**. Liquid UV-Vis spectroscopy was used for concentration change detection. Compound **6** can easily dissolve in aqueous solution; therefore, its adsorption capacity is not considered here. Figure 5 and Supplementary Figures S15–S19 show the standard diluted MB concentration changes along with the adsorption time in the presence of compounds **1**–**5**. The removal efficiency is very high for all the reported materials. As showed in Figure 5, compounds **2**–**5** adsorbed over 80% of MB in the first 10 min, and the adsorption process of compounds **2**–**5** was complete in no more than 2 h. However, compound **1** showed a poor removal efficiency of 10% in an hour due to lack of an exchangeable cation source and steric hindrance. IR spectra of the final recycled fine crystals confirmed that the process is an adsorption process by showing characteristic peaks of MB (1105 cm^−1^ and 1430 cm^−1)^ and **1**–**5**, respectively (Supplementary Figures S20–S24).

To fully to evaluate the specific capacity of MB, 15 mg of POMs were added to 100 mL MB solution at a MB concentration of 10 mg L^−1^ under stirring at room temperature. The solutions were separated from the adsorbent by means of centrifugation at regular time intervals, and 5 mL of sample solution was taken out and analyzed on a UV/Vis spectrophotometer. The dye adsorption amount q_t_ (mg g^−1^) was calculated by:

q_t_ = (C_0_ − C_t_) V/W (1)

where, C_0_ and C_t_ (mg L^−1^) are the liquid phase concentration of dyes at the beginning and after a given time t (min), respectively V (L) is the volume of the solution, and W (g) is the mass of the sample used. Compounds **1**–**5** possesses saturation capacity of 174.3 mg/g, 488.2 mg/g, 521.7 mg/g, 319.7 mg/g and 425.8 mg/g for the uptake of MB^+^ (Table 1) in 2 hours, separately. Among these complex hybrids, compound **3** is the most promising adsorbent candidate with the highest specific capacity and removal efficiency.

Aside from MB, cationic dyes like RhB^+^ with different sizes and anionic dyes like MO^−^ are usually the models used for selective adsorption and separation ability evaluations of this type of adsorbent. As shown in Supplementary Figures S26–S45, the selectivity is quite clear in that the compounds can selectively capture MB^+^ molecules from the corresponding binary mixtures. After 2 hours, the characteristic MB^+^ peak at 665 nm in the UV/Vis absorption spectrum almost disappears, while those of RhB^+^ (554 nm) or MO^−^ (475 nm) remain with slightly decreased intensity. Additionally, the reusability and stability of these complexes was also investigated. When these complexes were immersed in mixed solution of dichloromethane and dimethylamine hydrochloride, and the dyes were gradually released within 0.5 h. As shown in Supplementary Figure S46, from the desorption process of compounds 2 and 3, we speculated that dimethylammouium ion may have replaced MB^+^ as cationic part and a small amount of MB^+^, so at the second and third time, it still maintained good adsorption performance. When compounds **4** and **5** are desorbed, only a part of MB can be replaced by dimethylammouium ions, and some MB cannot be desorbed, which results in a decrease in performance. The structural integrities of the regenerated adsorbents were confirmed by PXRD (Supplementary Figures S47–S51); and the reasonable consistency of the patterns after three cycles with the simulated patterns indicates their good stability. Partial major peaks shifted indicated the adsorption process are not simple molecular guest exchange processes in the cavities and the complex cations in the framework was also involved may through protonation/deprotonation. Cation exchanges alternated the component packing mode and form supramolecular nano-aggragates in the solution.

The adsorption abilities of the compounds are obviously related to the structures and compositions of both the compounds and the dye molecules. In order to verify the electrostatic attraction between MB^+^ and the polyoxometalate units, solid NaCl was added at the beginning of adsorption process and the adsorption rate and special adsorption capacity decreased significantly (Supplementary Figure S52). This result indicates that an electrostatic attraction was produced between positively charged MB^+^ and POMs [82,83].

Interestingly, the significant different dye adsorption ability of the complexes demonstrated the advantage of these nanohybrids for new complex absorbent applications. Firstly, previous studies have corroborated this kind of adsorption are ion exchange process. This study also confirms that the exchangeable cations and available channel are key factors in improving the absorption ability. Secondly, considering the stability of the adsorbent, the additional releasable proton in the structure could further increase the absorption capacity. Finally, the transition metals in the complex do affect the absorption volume, and while they have showed very similar structural coordination behavior, the thermal analyses of compounds **2** and **3** confirm their stability difference, which could affect the ion-exchange processes. Consequently, compound **3** has shown the best absorption ability of MB^+^ so far, to the best of our knowledge. DMA in compounds **2** and **3** can be substituted by MB^+^ cations, as well as the proton on the complex cations [84]. The final data shows it has an adsorption molar ratio to dye and adsorbent of about 3:1. In compounds **1, 4** and **5**, only the proton on the imidazole N can be substituted and some of them are well fixed by H-bonding, and they lack exchangeable DMA cations which leads to an adsorption molar ratio decrease to 1: 1, 2:1 and 2:1, respectively.

## 3. Materials and Methods

All reagents purchased were used without further purification. X-ray diffraction (XRD, Rigaku, SmartLab, Tokyo, Japan) was employed to characterize compounds **1**–**6** using Cu-Kα in the range of 10–50° with the scanning rate of 10° min^−1^. The IR spectra were recorded with a Thermo Nicolet Corporation FTIR spectrometer (AVATR-360, Madison, WI, USA) in the 400–4000 cm^−1^ region with a KBr pellet. Thermogravimetric analyses (TGA) were performed on a PerkinElmer TG-7 (TA SDT Q600 instrument, Newcastle, Delaware, USA)) under a flowing Ar atmosphere with a heating rate of 10 °C min^−1^. The UV-vis spectra were obtained on a SHIMADZU UV-VIS spectrophotometer (UV-2600, Kyoto, Japan).

The crystallographic data and structural refinements for **1–6** are given in Tables S1 and S2. Single crystal X-ray analysis was performed on a Bruker APEX-II diffractometer (Smart 1000 CCD, Berlin, Germany) with graphite-monochromated Mo-Kα radiation at 296 K (λ = 0.71073 Å). A multi-scan technique was used for absorption correction using the Wingx program. Structures were solved with the ShelXT 2014 structure solution program using intrinsic phasing and refined with the ShelXL refinement package using least squares minimization. All of the non-hydrogen atoms were refined anisotropically on F^2^ by the full-matrix least-squares technique. The hydrogen atoms except those of solvent molecules were generated geometrically and refined isotropically using the riding model. For these complexes, some hydrogen atoms attached to solvents were not located, but were included in the structure factor calculations. In complex **1**, the unit cell includes some region of disordered solvent molecule, which could not be modeled as discrete atomic sites. Thus, the SQUEEZE routine of PLATON was applied to remove the contributions to the scattering from the solvent molecules. The reported refinements are of the guest-free structures using the ⁄.hkp files produced by using the SQUEEZE routine. In the refinement of complex **3**, the restrains command ISOR and DELU was used on N14 and C12 to avoid the NDP problem. During the refinement of complex **5**, the commands DFIX, ISOR and DELU were used to restrain one DMF molecule. The command ISOR and DELU were used to restrain one aromatic ring of the organic ligand. The amount of solvent molecules of complexes came from the TG experiment. The details of the crystal parameters, data collection and refinements for complexes **1**–**4** are summarized in Tables S1 and S2. CCDC 1866410, 1866412, and 1866416–1866419 contain the supplementary crystallographic data for this paper.

### 3.1. Synthesis of 2,2′-Biimidazole Ligand

The 2,2′-biimidazole ligand was synthesized according to a reported procedure [83]. In a typical reaction, aqueous glyoxal (23.0 mL, 40 wt%) was added dropwise to a vigorously stirred solution of ammonium acetate (70.00 g) and H_2_O (23.0 mL) at room temperature over a period of 4 h. After completion of the reaction, the product was filtered and washed several times of water and acetone. δ^H^ ([D_6_] DMSO) 7.08 (d, 4H), 12.68 (s, 2H).

### 3.2. Synthesis of [Ni(H_2_biim)_3_]_2_[Mo_8_O_26_]•8DMF (**1**)

MoO_3_ (3.00 g, 20.83 mmol), H_2_biim (0.20 g, 1.49 mmol), and Ni(CH_3_COO)_2_•4H_2_O (0.12 g, 0.48 mmol) were added to 50.0 mL aqueous DMF solution (H_2_O/DMF = 10/40), and then the solution was stirred at 120 °C for 4 h and was filtered. Deep purple crystals appeared after about one day of slow evaporation at room temperature. Yield: 42% Anal. *Cal*.: C, 26.81; H, 3.4; N, 16.66. Found as follows: C, 27.15; H, 3.81; N, 16.05. IR (KBr, cm^−1^): 3446 (br), 2410 (w), 1653 (s), 1508 (w), 1428 (m), 1370 (m),1323 (w), 990 (vs), 950 (vs), 850 (m), 550 (w), 521 (w).

### 3.3. Synthesis of (DMA)_2_[Ni(H_2_biim)_2_(H_2_O)_2_][Mo_8_O_26_]•4DMF (**2**)

MoO_3_ (3.00 g, 20.83 mmol), H_2_biim (0.20 g, 1.49 mmol), and Ni(CH_3_COO)_2_•4H_2_O(2.00 g, 8.04 mmol) added to 50.0 mL mixture solution (H_2_O/DMF = 15/35). The solution was stirred at 120 °C for an additional 4 h and was filtered. Green crystals appeared after about two days of slow evaporation at room temperature. Yield: 30%. Anal. *Cal.*: C, 17.40; H, 3.10; N, 10.15. Found as follows: C, 17.93; H, 3.21; N, 9.97. IR (KBr, cm^−1^): 3352 (br), 1657 (vs), 1531 (W), 1457 (w), 1386 (m),1000 (m), 947 (vs), 913 (s), 846 (s), 709 (m), 557 (w), 523 (w).

### 3.4. Synthesis of (DMA)_2_[Co(H_2_biim)_2_(H_2_O)_2_][Mo_8_O_26_]•4DMF (**3**)

MoO_3_ (2.24 g, 15.56 mmol), H_2_biim (0.20 g, 1.49 mmol), and Co(CH_3_COO)_2_•4H_2_O(1.04 g, 4.17 mmol) added 50.0 mL of aqueous DMF (H_2_O/DMF = 15/35), and then the solution was stirred at 100 °C for 4 h and filtered. Pink crystals appeared after about three days of slow evaporation at room temperature. Yield: 30% Anal. *Cal.*: C, 17.40; H, 3.11; N, 10.15, Found as follows: C, 17.68; H, 3.05; N, 10.23. IR (KBr, cm^−1^): 3358 (br), 2928(w), 1659 (vs), 1530 (s),1387 (m), 1107 (s), 947 (vs), 845 (vs), 770 (s),557 (w),522 (w).

### 3.5. Synthesis of [Zn(H_2_biim)(DMF)_3_]_2_[Mo_8_O_26_]•2DMF (**4**)

MoO_3_ (3.00 g, 20.83 mmol), H_2_biim (0.20 g, 1.49 mmol), and Zn(CH_3_COO)_2_•2H_2_O(1.00 g, 4.56 mmol) added to 50.0 mL of aqueous DMF (H_2_O/DMF = 15/35). The solution was stirred at 100 °C for 4 h and was filtered. Colorless crystals appeared in about one day of slow evaporation at room temperature Yield: 37%. Anal. *Cal.*: C, 19.94; H, 3.14; N, 10.34 Found as follows: C, 19.72; H, 3.05; N, 10.05. IR (KBr, cm^−1^): 3500 (br), 1657 (vs),1438 (w), 1385 (m), 1110 (m), 949 (s), 915 (s), 842 (s), 775 (w), 560 (w), 520 (w).

### 3.6. Synthesis of [Cu(H_2_biim)(DMF)_3_]_2_[Mo_8_O_26_]•2DMF (**5**) and (DMA)_2_[Cu(DMF)_4_][Mo_8_O_26_]•2DMF (**6**)

MoO_3_ (2.24 g, 15.56 mmol), H_2_biim (0.20 g, 1.49 mmol), and Cu(CH_3_COO)_2_•H_2_O (1.00 g, 5.01 mmol) added to 50.0 mL of aqueous DMF (H_2_O/DMF = 10/40). The solution was stirred at 100 °C for 4 h and was filtered. Green crystals of **5** and blue-green block crystals of **6** appeared after about two days of slow evaporation at room temperature. The two types of crystals were separated manually. Yield of 5: 15%. Anal. *Cal.*: C, 19.97; H, 3.14; N, 10.35 F Found as follows: C, 19.06; H, 3.02; N, 10.0. IR (KBr, cm^−1^): 3446 (br),3130 (w), 1648 (vs), 1525 (m),1430 (m), 947 (s),913 (s), 845 (m), 557 (w), 523 (w). yield of 6: 52%. Anal. *Cal*.: C, 14.85; H, 3.26; N, 6.30. Found as follows: C,14.94; H, 3.30; N, 6.28. IR (KBr, cm^−1^): 3446 (br),1645 (vs), 1367 (s),1254 (m),1120 (m), 943 (vs), 907 (vs), 715 (s), 557 (m), 523 (m)

## 4. Conclusions

A series of new POM-complex hybrid compounds based on β-[Mo_8_O_26_]^4−^ cluster and H_2_biim organic ligands have been successfully synthesized and characterized. The addition of the organic ligand affects the solid structure, and therefore changes the dye adsorption properties of these new compounds. The results indicate that they are good, rapid and effective adsorbents for dye separation. Compound **3** has the highest capacity to adsorb MB^+^ reported so far, and it shows excellent reusability, stability and ability to selectively capture MB^+^ from binary-mixture solutions of MB^+^/MO^−^ and MB^+^/RhB^+^. The discovery on their structure and relevant adsorbent behavior not only adds a representative architecture to the limited family of POM-complex hybrid, but may also motivate researchers to develop unprecedented hybrid and improve their adsorbent applications.

## Figures and Tables

**Table 1:** Comparison of MB^+^ adsorption capacity in various materials.

Materials	Adsorption Capacity (mg/g)	Time (hrs)	Ref
Zn-DDQ ^a^	135	8	[77]
C_12_H_64_As_2_Cu_3_N_12_O_69_W_18_^b^	320.5	1.5	[78]
MWCNTs/Fe_2_O_3_^c^	42.3	1	[79]
graphene oxide sponges	397	1	[80]
PW_11_V@MIL-101 ^d^	371	0.5	[67]
ErCu–POM (Er-3) ^e^	391.3	0.5	[81]
Compound 1	174.3	2	This work
Compound 2	488.2	2	This work
Compound 3	521.7	2	This work
Compound 4	425.8	2	This work
Compound 5	319.7	2	This work

^a^ [Zn(DDQ)(H_2_O)_2_]·2H_2_O·DMF (H_2_DDQ = N,N′-dibenzoic acid 2,3-diaminoquinoxaline). ^b^ C_12_H_64_As_2_Cu_3_N_12_O_69_W_18_ = [Cu(en)_2_(OH_2_)]_2_{Cu(en)_2_}{As_2_W_18_O_61_}]·6H_2_O. ^c^ MWCNTs = multi-walled carbon nanotubes. ^d^ PW_11_V = K_4_PW_11_VO_40._^e^ ErCu-POM = [Cu(en)_2_]_3_[Er(H_2_O)SiW_11_O_39_]_2_(C_2_O_4_)[Cu(H_2_O)(en)_2_]_3_.

## Supplementary Materials

Supporting information for this article is given via a link at the end of the document.
