# Supplementary material for: A Comprehensive Study on the Dye Adsorption Behavior of Polyoxometalate-Complex Nano-Hybrids Containing Classic β-Octamolybdate and Biimidazole Units
Source: Molecules. 2019 Feb 22;24(4):806. doi: 10.3390/molecules24040806 (PMC6412703; doi:10.3390/molecules24040806)
Supplement: Supplementary file 1 [file molecules-24-00806-s001.pdf]

Supporting Information

A Comprehensive Study on the Dye Adsorption Behavior of Polyoxometalate-Complex Nano-Hybrids Containing Classic β-Octamolybdate and Biimidazole Units

Shuang Liang [1], Yan-Mei Nie [1], Sang-Hao Li [1], Jian-Liang Zhou [1] and Jun Yan [2,*]

[1] School of Chemistry and Chemical Engineering, Central South University, Changsha 410083, China; liang324@csu.edu.cn (S.L.); 15367493565@163.com (Y.-M.N.); 172311031@csu.edu.cn (S.-H.L.); zhoujl@csu.edu.cn (J.-L.Z.)

[2] Hunan Provincial Key Laboratory of Efficient and Clean Utilization of Manganese Resources, Central South University, Changsha 410083, Hunan, China

* Correspondence: yanjun@csu.edu.cn; Tel./Fax: +86-731-8887-9616

Table of Contents

Table S1-S3 Crystallographic data

Figure S1-S2 solid packing data of the compounds

Figure S3-S8 Simulated and experimental XRD spectra of compounds

Figure S9-S14 the TG curves of compounds

Figure S15-S19 Complexes was used to adsorb MB aqueous solution

Figure S20-S25 The IR spectra of compounds.

Figure S26-S30 The selective adsorption capability of complexestoward the mixed dyes: RhB and MB.

Figure S31-S35 The selective adsorption capability of compound 1 toward the mixed dyes: MO and MB.

Figure S36-S40 Complexes was used to adsorb RhB aqueous solution

Figure S41-S45 Complexes was used to adsorb MO aqueous solution

Figure S46 Recycling tests of Compound 2-5 toward MB adsorption

Figure S47-S51 Experimental and after adsorption XRD pattern of compounds

Fig. S52: Compound 3 was used to adsorb MB+NaCl aqueous solution

1. **Crystallographic data**

Table S1: Crystallographic Details for Compound **1**to**3**

Compound	**1**	**2**	**3**
Empirical formula	$C_{60}H_{92}Mo_8N_{32}Ni_2O_{34}$	$C_{28}H_{60}Mo_8N_{14}NiO_{32}$	$C_{28}H_{60}CoMo_8N_{14}O_{32}$
CCDC number	1866410	1866412	1866416
Formula weight	2690.38	1931.13	1931.35
Temperature (K)	296(2)	296(2)	296(2)
Crystal system	monoclinic	Triclinic	Triclinic
Space group	$P2_1/c$	P-1	P-1
a (Å)	14.6105(10)	10.7023(2)	10.6880(2)
b (Å)	12.2808(8)	14.9152(3)	14.9465(3)
c (Å)	25.7996(16)	19.0214(3)	19.0881(3)
α (°)	90	83.7490(10)	83.8750(10)
β (°)	97.184(4)	79.6160(10)	79.7210(10)
γ (°)	90	82.5880(10)	82.5600(10)
V ($Å^3$)	4592.8(5)	2950.21(9)	2964.15(9)
Z	2	2	2
Calculated density(g/cm^3)	1.840	2.174	2.164
μ (mm^{-1})	1.543	2.050	2.003
θ (°)max	27.612	25.999	25.999
F (000)	2426.0	1892.0	1890.0
Reflections collected	45925	41858	34642
Independent reflections	10541	11563	11628
R_{int}	0.0687	0.0203	0.0257
Goodness-of-fit on F^2	0.966	1.205	1.074
Final *R* indices[$I>2\sigma(I)$]	0.0553	0.0325	0.0318
wR_2 (all data)	0.1365	0.0759	0.0678

Table S2: Crystallographic Details for Compound **4** to6

Compound	**4**	**5**	**6**
Empirical formula	$C_{36}H_{68}Mo_8N_{16}O_{34}Zn_2$	C_{36} $H_{68}Cu_2Mo_8N_{16}O_{34}$	$C_{22}H_{58}CuMo_8N_8O_{32}$
CCDC number	1866417	1866418	1866419
Formula weight	2166.66	2163.66	1777.82
Temperature (K)	296(2)	296(2)	296(2)
Crystal system	Triclinic	Triclinic	Triclinic
Space group	P-1	P-1	P-1
a (Å)	12.2734(3)	12.2778(3)	11.6324(4)
b (Å)	12.4298(3)	12.5496(3)	11.7465(5)
c (Å)	13.8568(3)	13.8876(4)	11.9228(5)
α (°)	66.0120(10)	66.945(2)	112.781(2)
β (°)	80.0070(10)	80.377(2)	104.649(2)
γ (°)	62.8400(10)	62.501(2)	107.411(2)
V (Å^3)	1718.21(7)	1746.22(9)	1301.24(9)
Z	1	1	1
Calculated density (g/cm^3)	2.087	2.057	2.269
μ (mm^{-1})	2.186	2.075	2.357
θ (°)max	26.000	26.000	25.998
F (000)	1064.0	1056.0	867.0
Reflections collected	22358	18901	15828
Independent reflections	6715	6815	5091
R_{int}	0.0170	0.0357	0.0308
Goodness-of-fit on F^2	1.215	1.041	1.159
Final *R* indices[I>2σ(I)]	0.0367	0.0600	0.0391
wR2 (all data)	0.0997	0.1704	0.0914

Table S3 selected bond length Å around the transition metal ions in the complexes.

	**Compound 1**		
Ni1-N1	2.100(5)	Ni1-N6	2.076(5)
Ni1-N5	2.121(5)	Ni1-N2	2.117(6)
Ni1-N12	2.103(5)	Ni1-N9	2.079(6)
	**Compound 2**		
Ni1-O27	2.082(5)	Ni2-O28	2.062(5)
Ni1-N5	2.122(4)	Ni2-N3	2.125(4)
Ni1-N6	2.078(4)	Ni2-N4	2.090(4)
	**Compound 3**		
Co1-O27	2.070(5)	Co2-O28	2.090(3)
Co1-N3	2.132(4)	Co2-N1	2.171(4)
Co1-N4	2.173(4)	Co2-N2	2.125(4)
	**Compound4**		
Zn1-O1	2.495(4)	Zn1-O16	2.047(3)
Zn1-O14	2.052(6)	Zn1-N7	2.109(5)
Zn1-O15	2.091(4)	Zn1-N8	2.108(4)
	**Compound 5**		
Cu1-O1	2.557(8)	Cu1-O18	2.303(9)
Cu1-O16	1.91(1)	Cu1-N1	2.076(9)
Cu1-O17	1.958(7)	Cu1-N2	1.99(1)
	**Compound 6**		
Cu1-O1	2.392(4)	Cu1-O15	1.955(5)
Cu1-O14	1.965(6)		

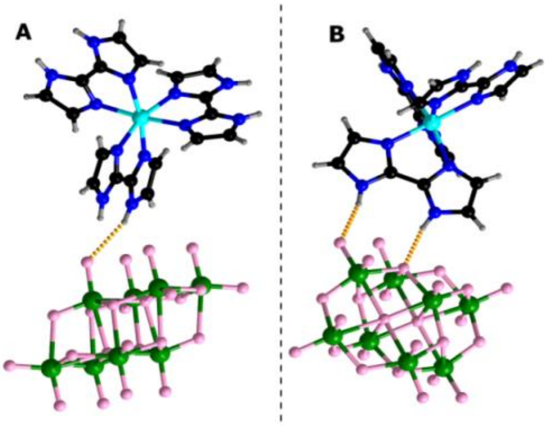

Fig. S1 The structural view of the two types of H-bond between POM and $[Ni(H_2biim)_3]^{2+}$ cation in compound **1**. The H-bond was shown in dashed line.

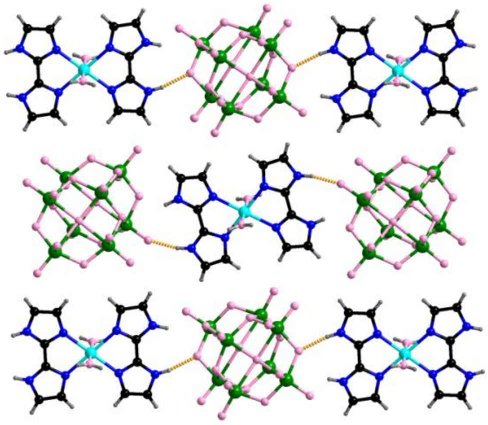

Fig.S2 The structural view of the two types of H-bond between POM and $[Ni(biim)_2(H_2O)_2]^{2+}$ cation in compound **2**. The H-bond was shown in dashed line. (color code: Mo-green; Ni – cyan; O – pink; N – blue; C –black; H - grey)

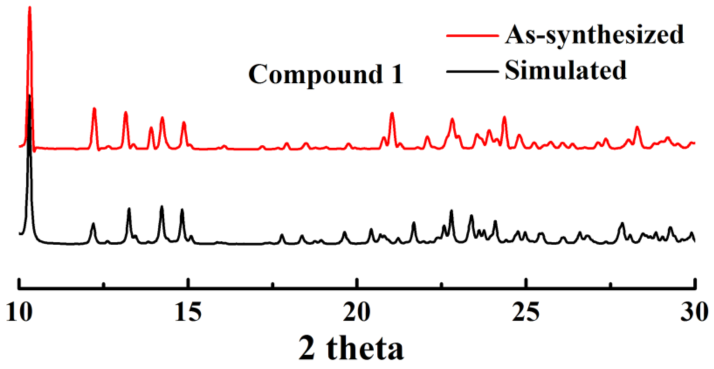

Fig. S3. Simulated and experimental XRD spectra of compound 1

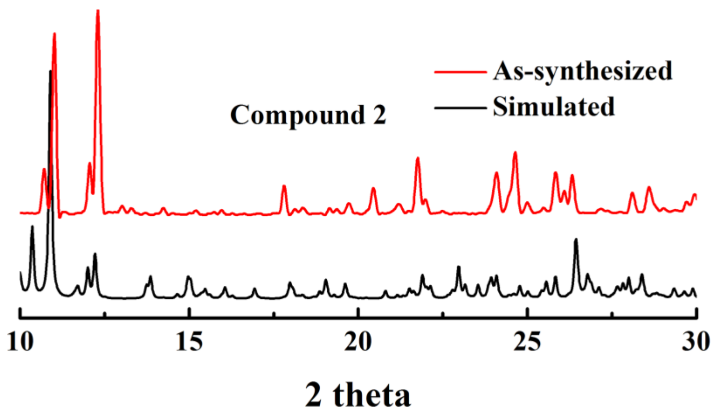

Fig. S4. Simulated and experimental XRD spectra of compound 2.Due to the moisture absorption of DMA cations and DMF, partial crystal structures decomposed

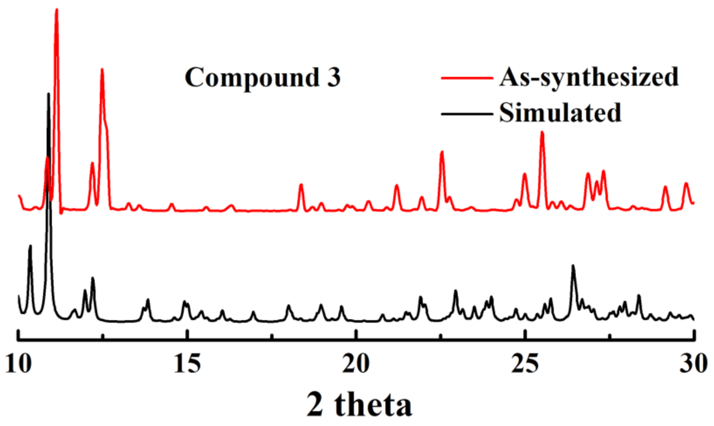

Fig. S5. Simulated and experimental XRD spectra of compound 3Due to the moisture absorption of DMA cations and DMF, partial crystal structures decomposed.

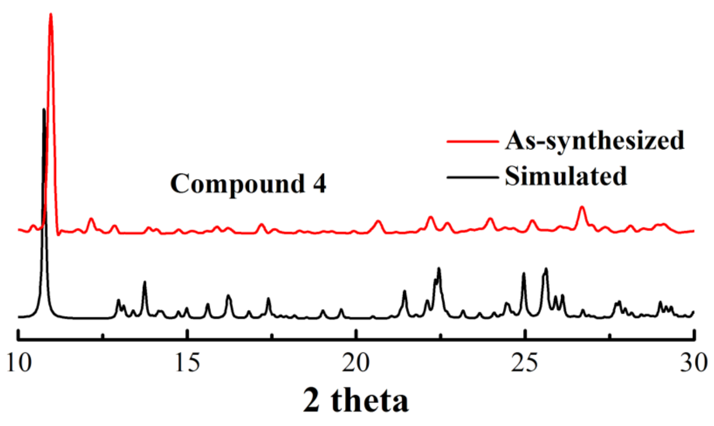

Fig. S6. Simulated and experimental XRD spectra of compound 4.Due to the moisture absorption of DMF, partial crystal structures decomposed.

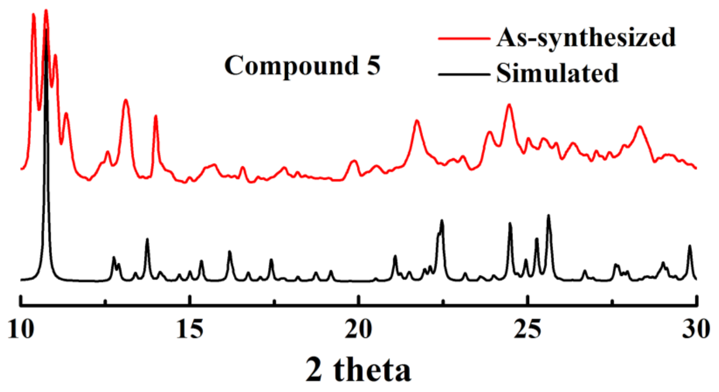

Fig. S7. Simulated and experimental XRD spectra of compound 5.Due to the moisture absorption of DMF, partial crystal structures decomposed.

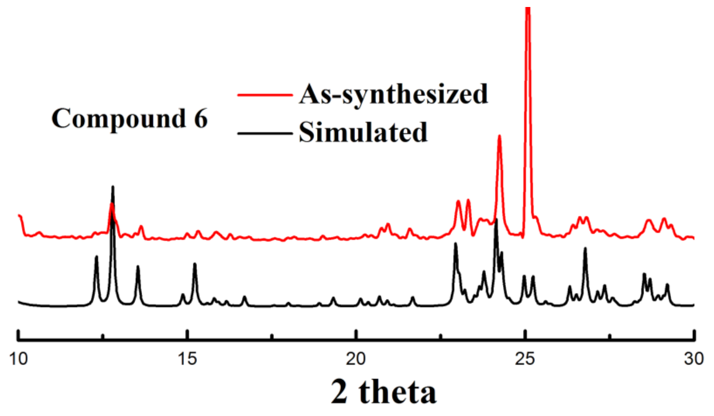

Fig. S8. Simulated and experimental XRD spectra of compound 6

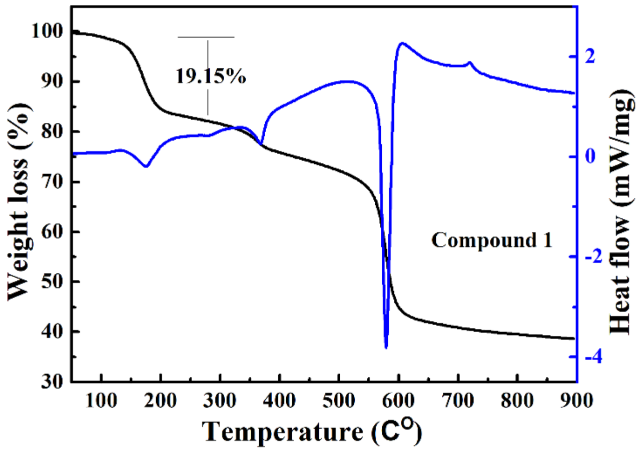

Fig. S9.The TG curves of compound 1

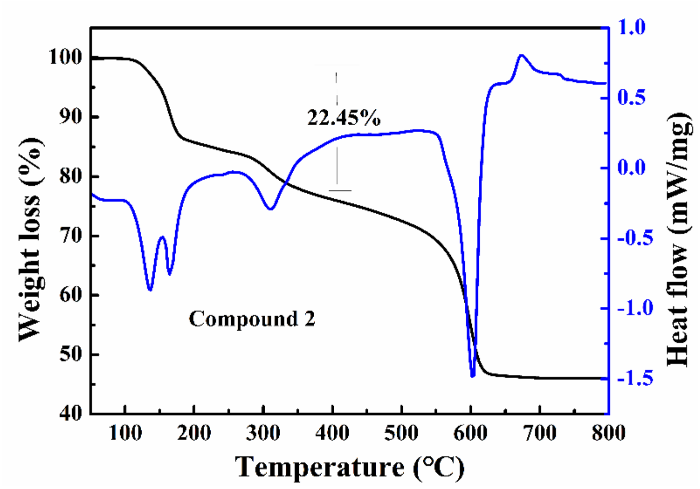

Fig. S10.The TG curves of compound 2

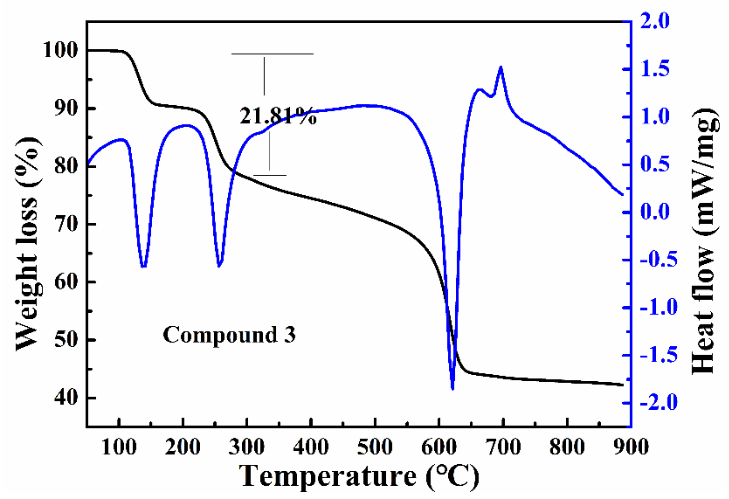

Fig. S11.The TG curves of compound 3

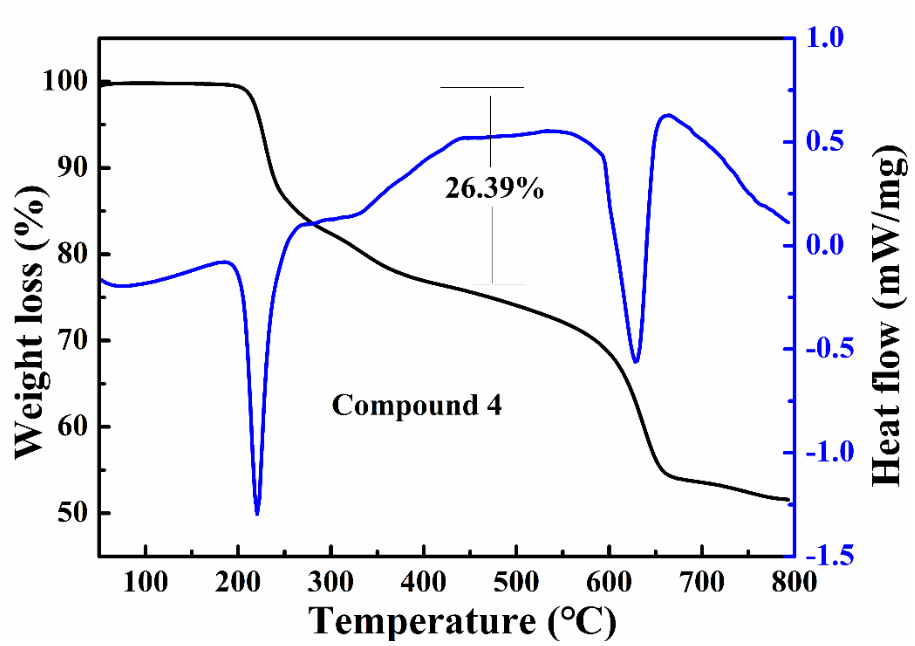

Fig. S12.The TG curves of compound 4

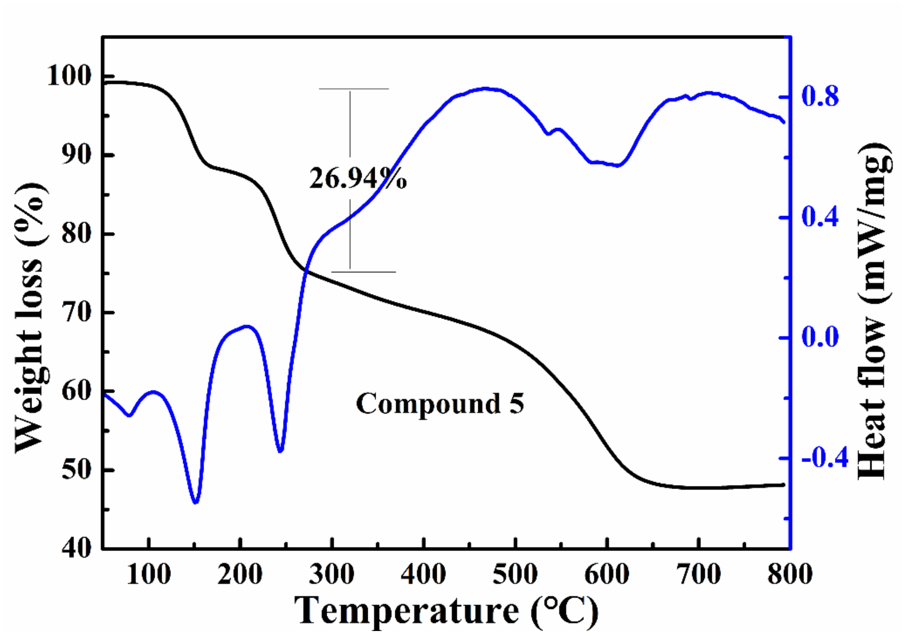

Fig. S13.The TG curves of compound 5

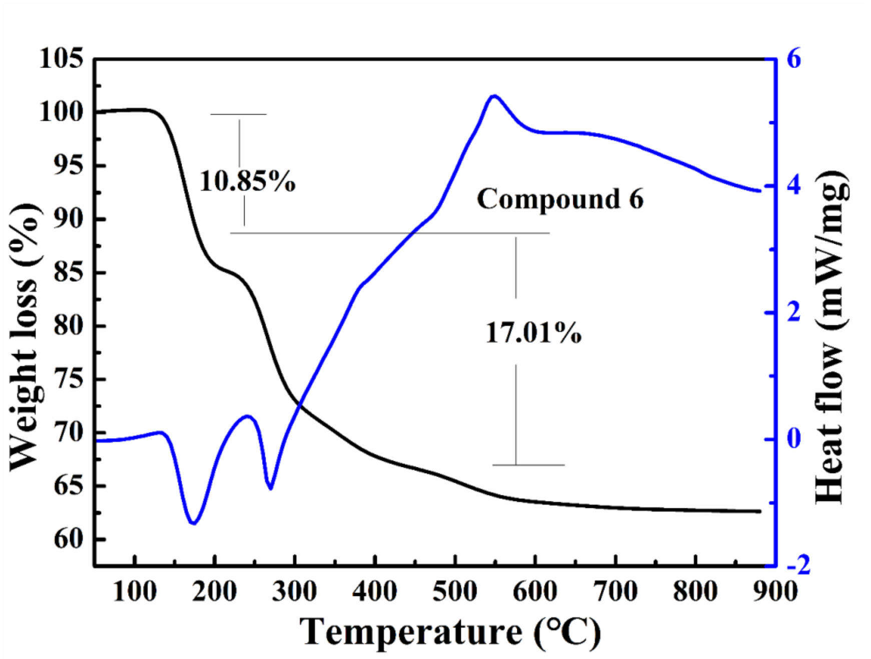

Fig. S14.The TG curves of compound 6

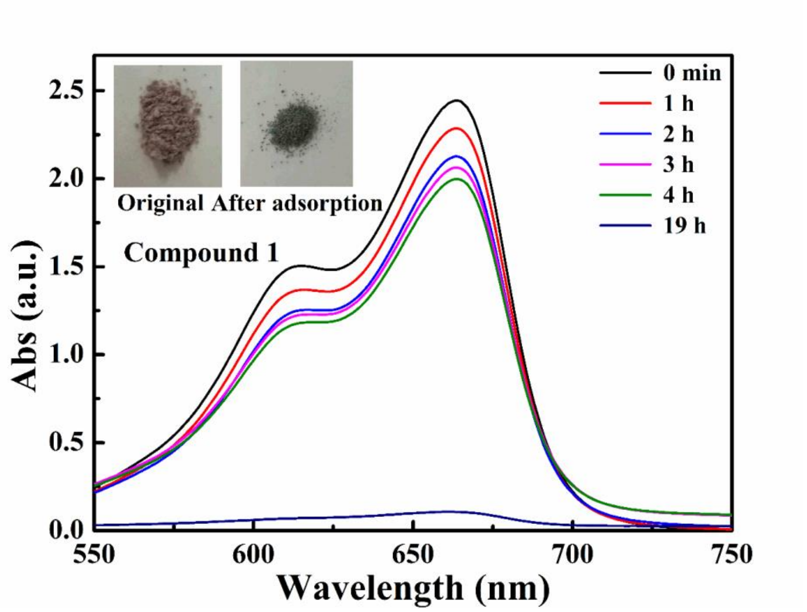

Fig. S15.Compound **1** was used to adsorb MB (10 mg/L, 100 mL) aqueous solution

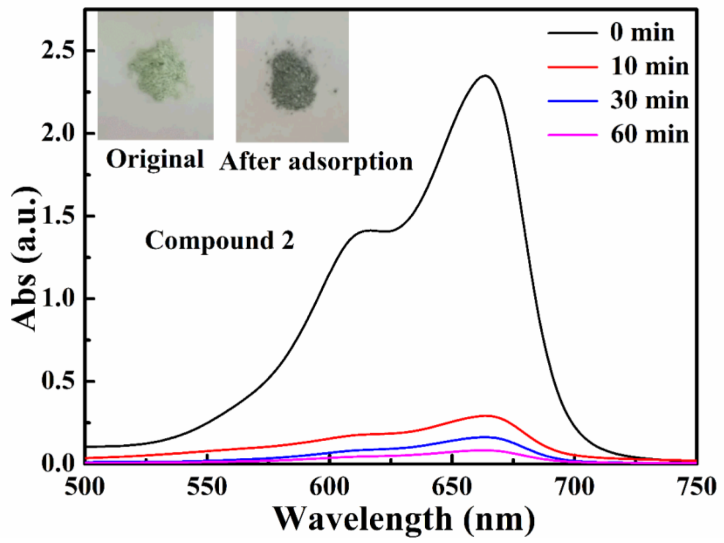

Fig. S16.Compound **2** was used to adsorb MB (10 mg/L, 100 mL) aqueous solution

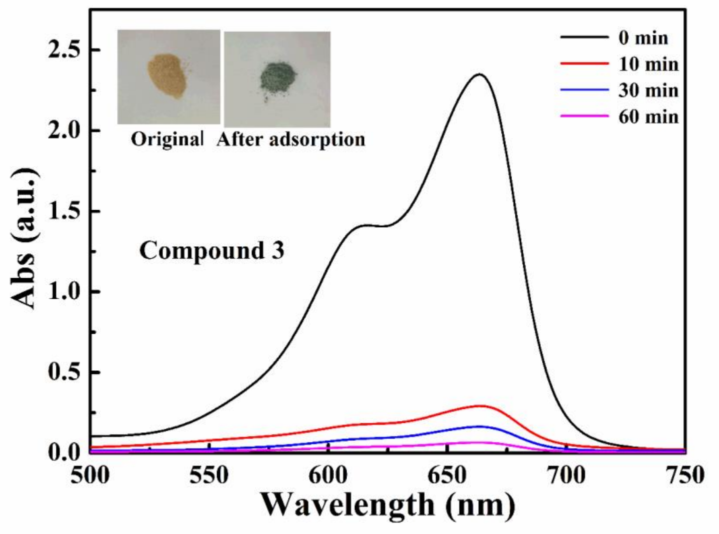

Fig. S17.Compound **3** was used to adsorb MB (10 mg/L, 100 mL) aqueous solution

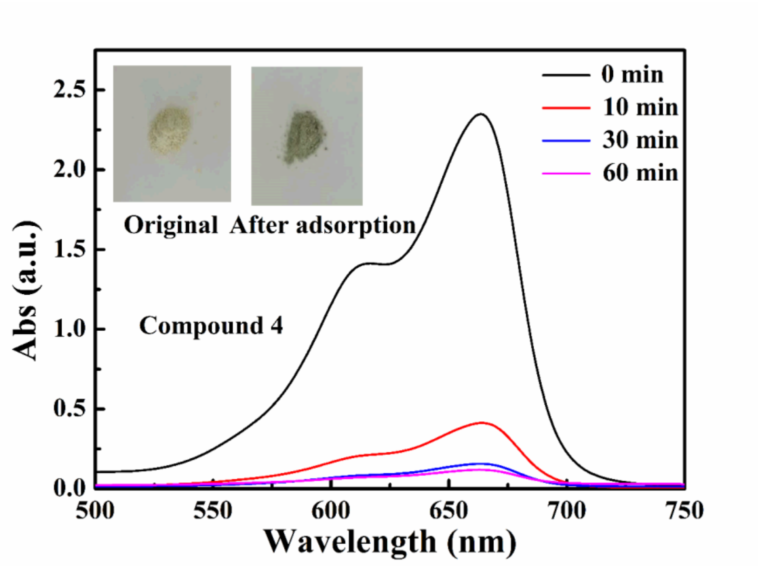

Fig. S18.Compound **4** was used to adsorb MB (10 mg/L, 100 mL) aqueous solution

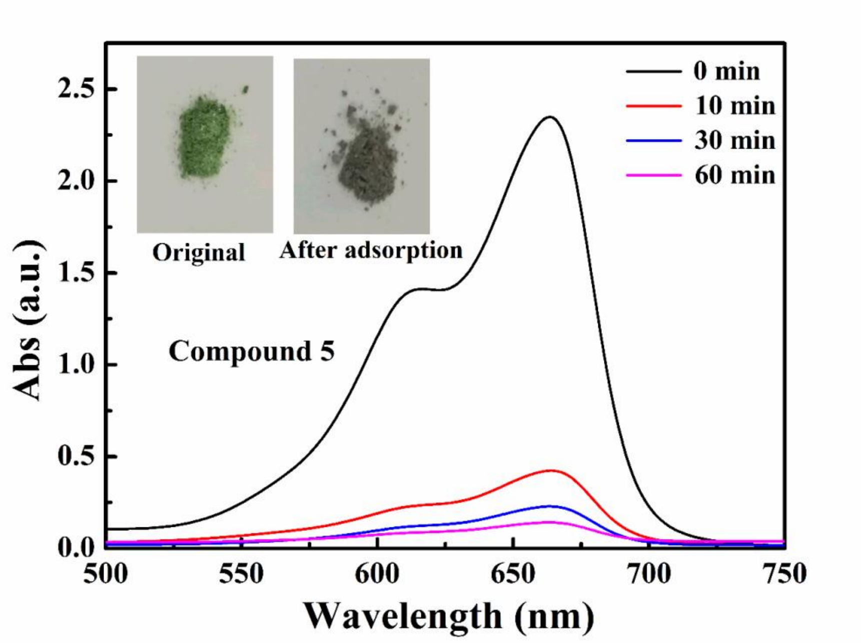

Fig. S19. Compound **5** was used to adsorb MB (10 mg/L, 100 mL) aqueous solution

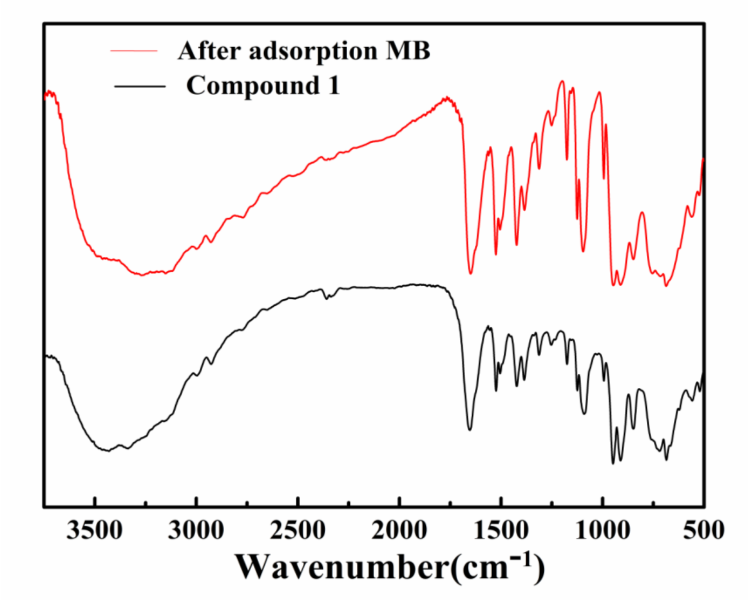

Fig. S20.IR spectra of the final recycled powdered solidand compound **1**.

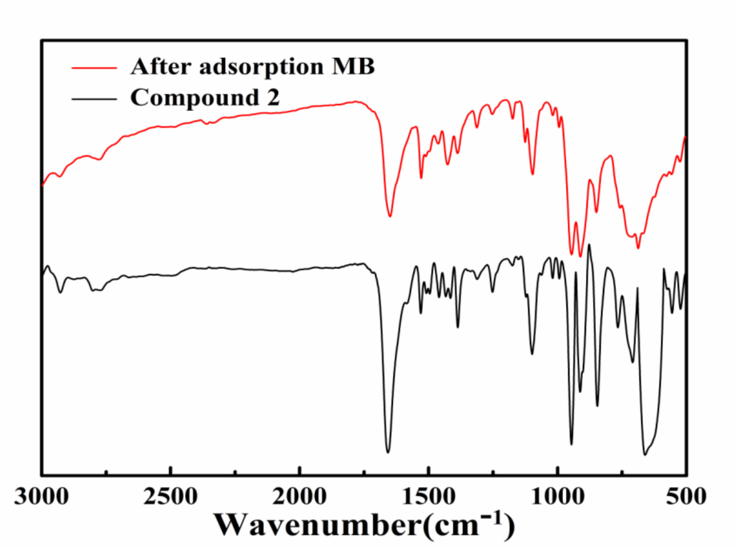

Fig. S21.IR spectra of the final recycled powdered solidand compound **2**

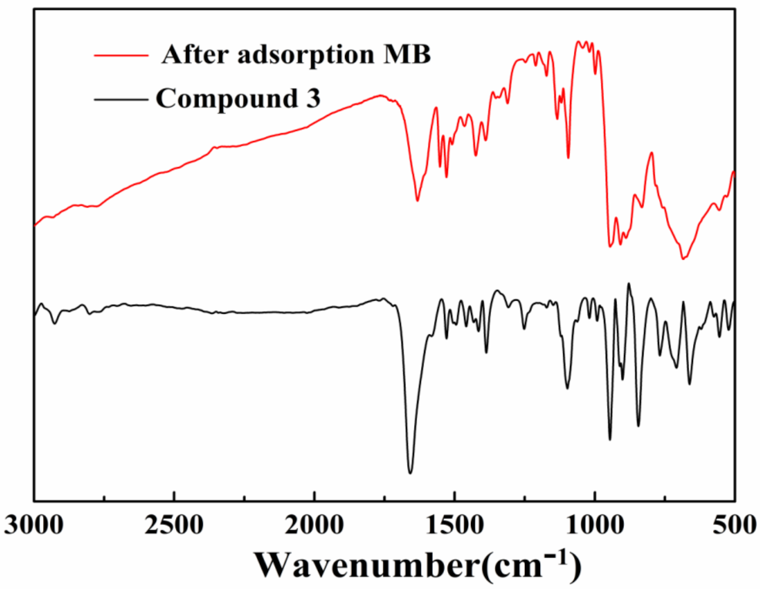

Fig. S22.IR spectra of the final recycled powdered solidand compound **3**.

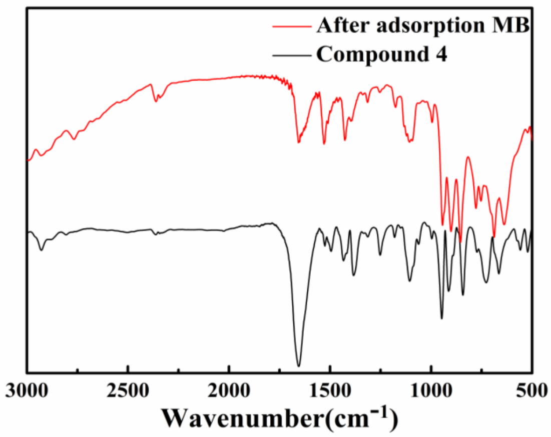

Fig. S23.IR spectra of the final recycled powdered solidand compound **4**

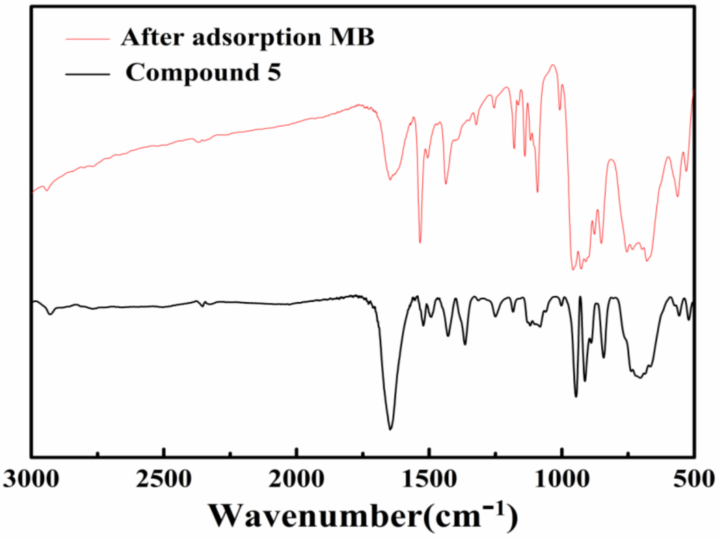

Fig. S24.IR spectra of the final recycled powdered solidand compound **5.**

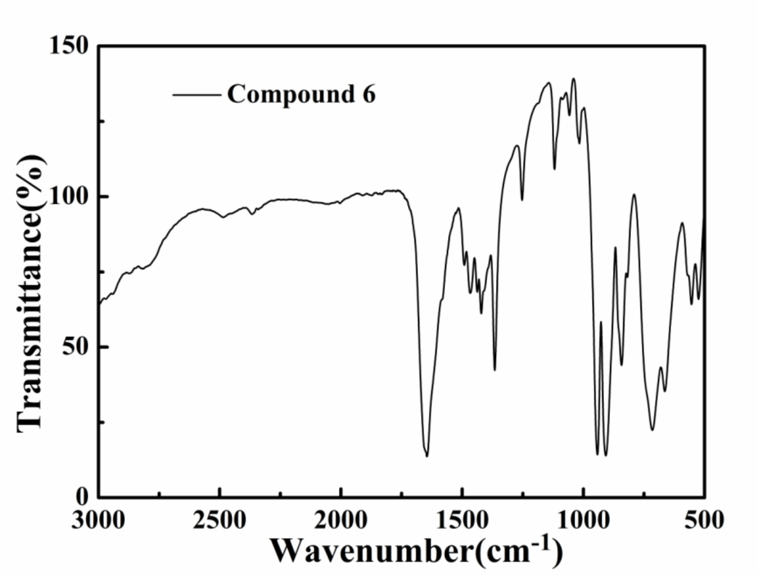

Fig. S25.IR spectra of Compound **6.**

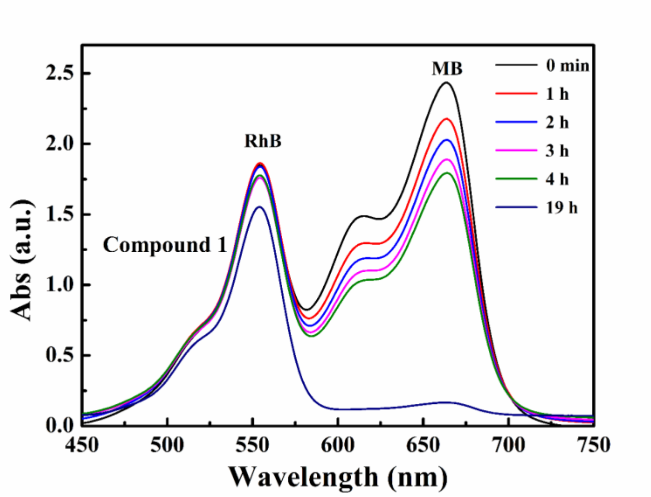

Fig. S26.The selective adsorption capability of compound **1** toward the mixed dyes: RhB and MB.

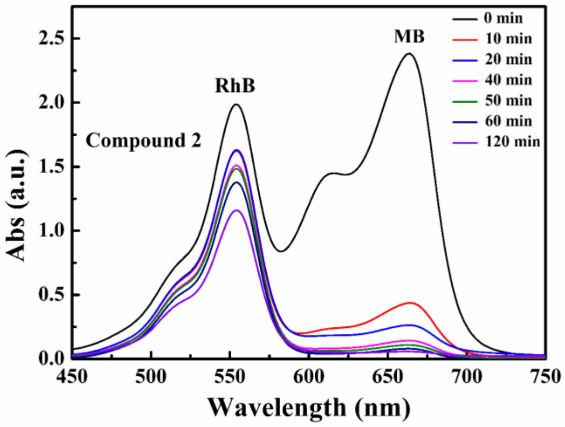

Fig. S27.The selective adsorption capability of compound **2**toward the mixed dyes: RhB and MB.

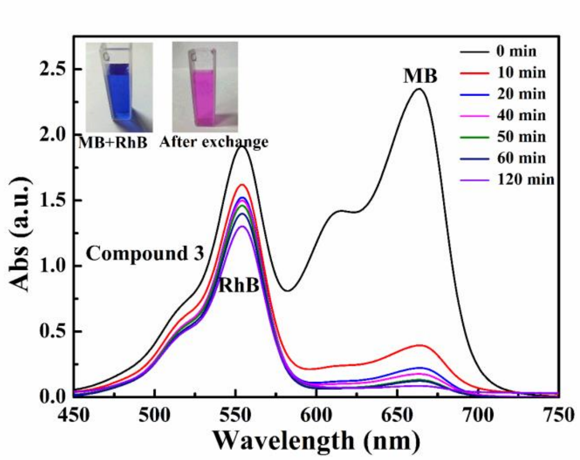

Fig. S28.The selective adsorption capability of compound **3**toward the mixed dyes: RhB and MB.

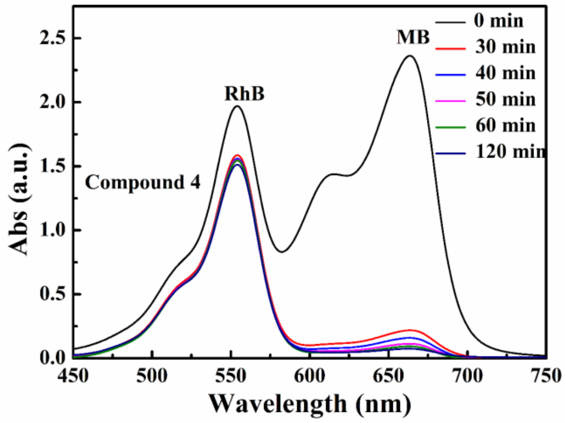

Fig. S29.The selective adsorption capability of compound 4toward the mixed dyes: RhB and MB.

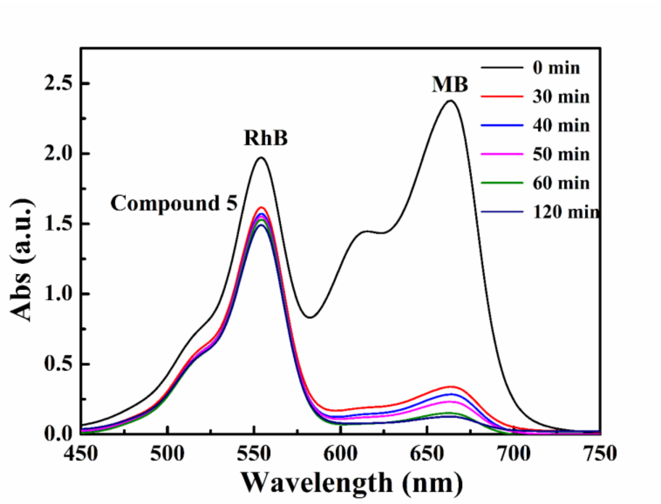

Fig. S30.The selective adsorption capability of compound **1** toward the mixed dyes: RhB and MB.

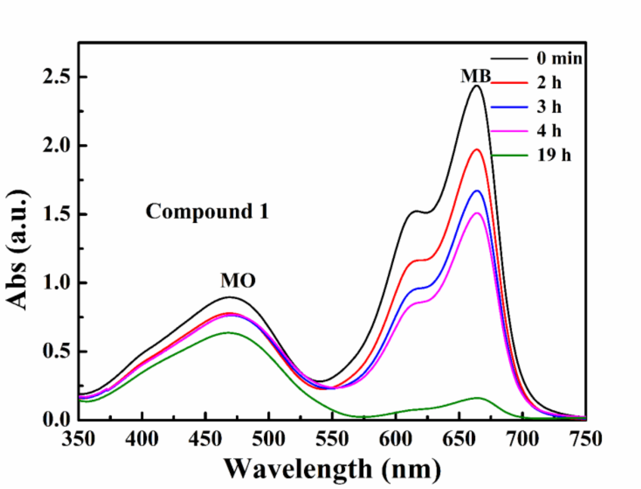

Fig. S31.The selective adsorption capability of compound **1** toward the mixed dyes: MO and MB.

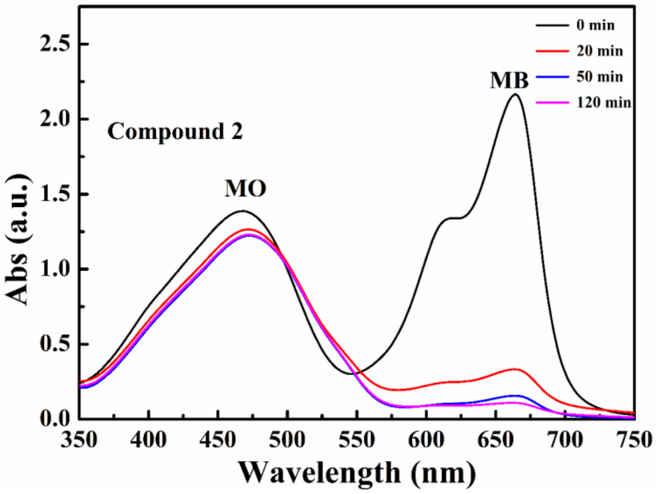

Fig. S32.The selective adsorption capability of compound **2** toward the mixed dyes: MO and MB.

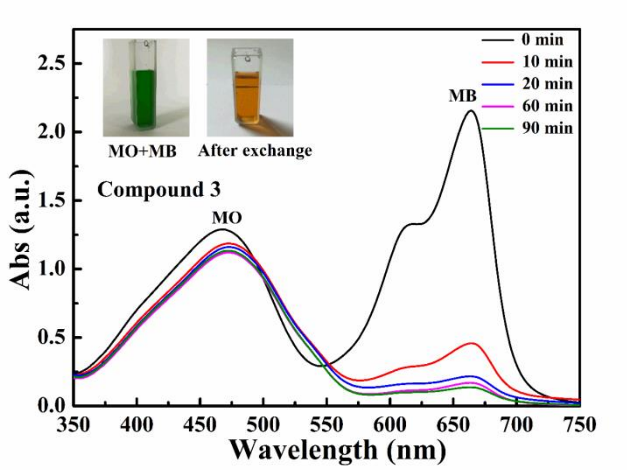

Fig. S33.The selective adsorption capability of compound **3** toward the mixed dyes: MO and MB.

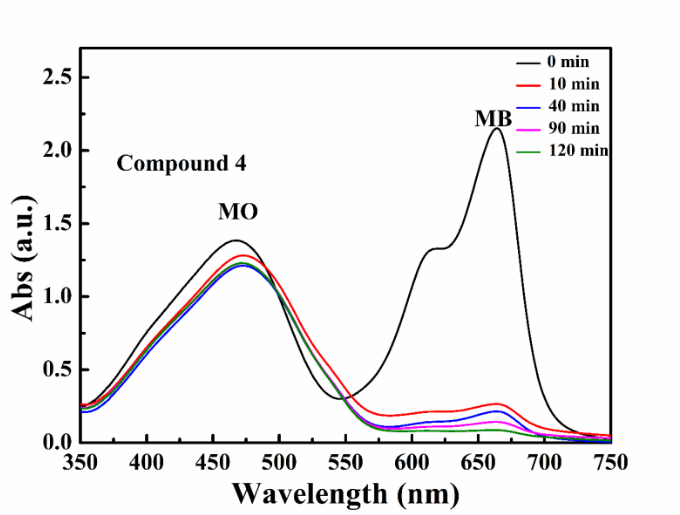

Fig. S34.The selective adsorption capability of compound **4** toward the mixed dyes: MO and MB.

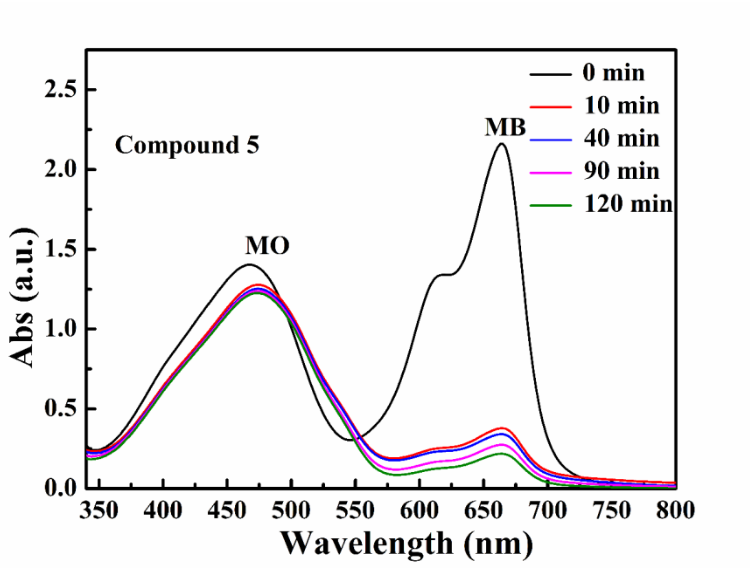

Fig. S35.The selective adsorption capability of compound **5** toward the mixed dyes: MO and MB.

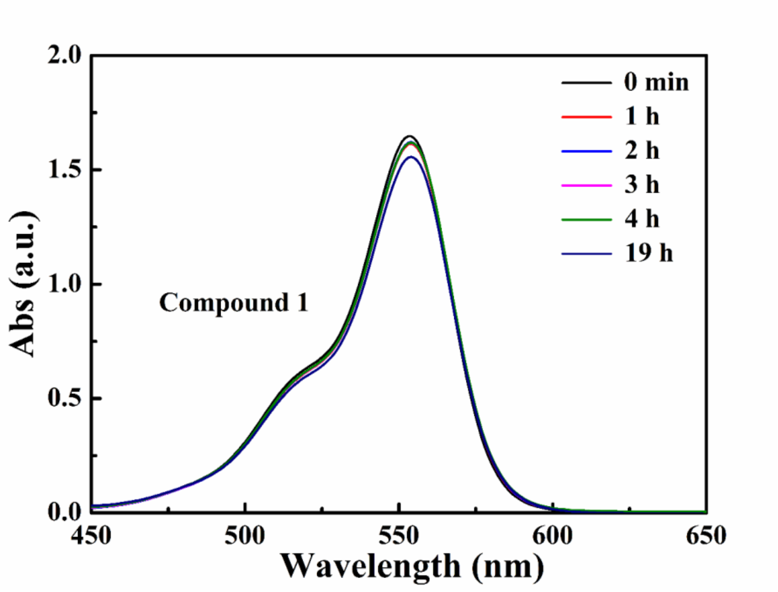

Fig. S36.Compound **1** was used to adsorb RhB aqueous solution

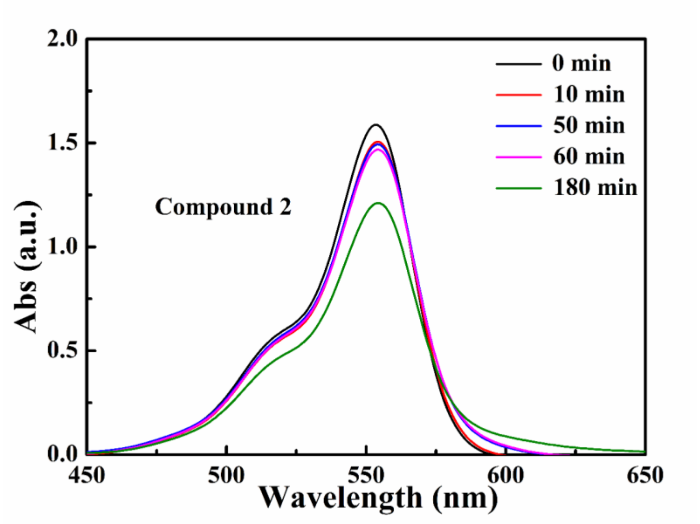

Fig. S37.Compound **2** was used to adsorb RhB aqueous solution

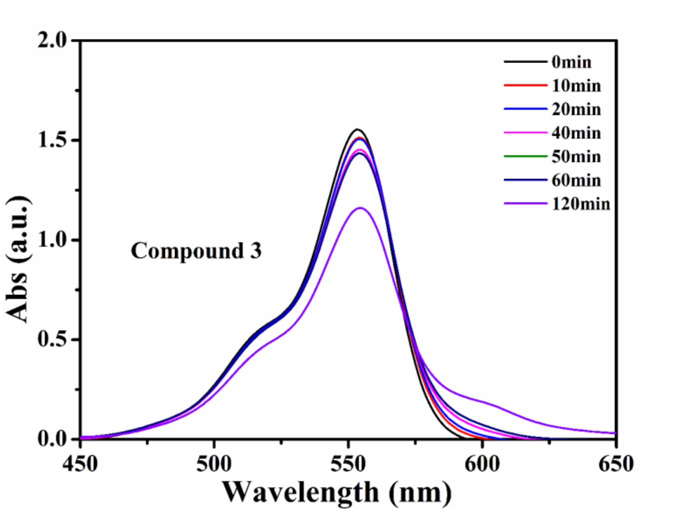

Fig. S38. Compound **3** was used to adsorb RhB aqueous solution

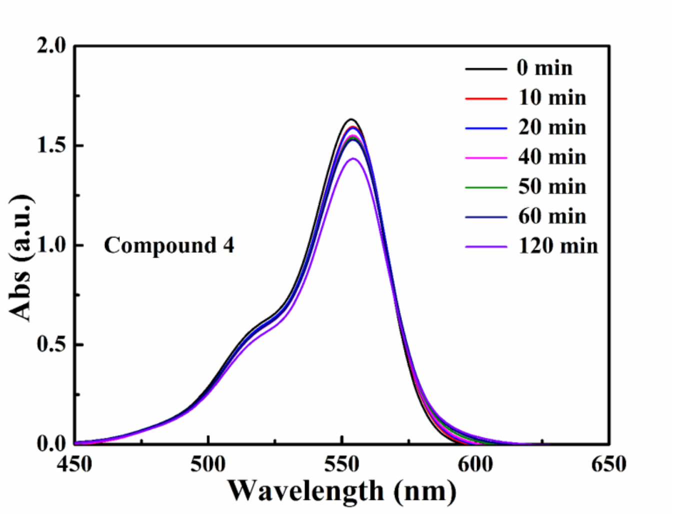

Fig. S39. Compound **4** was used to adsorb RhB aqueous solution

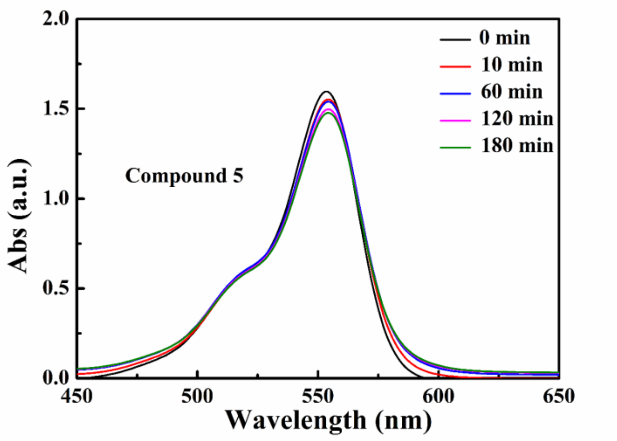

Fig. S40. Compound **5** was used to adsorb RhB aqueous solution

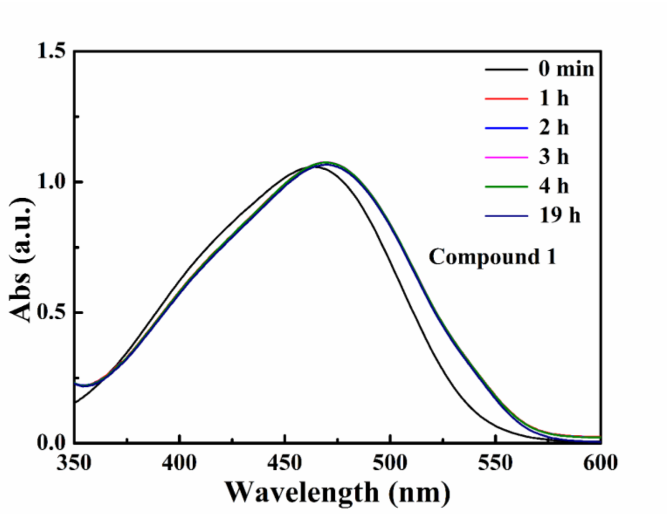

Fig. S41.Compound **1** was used to adsorb MO aqueous solution

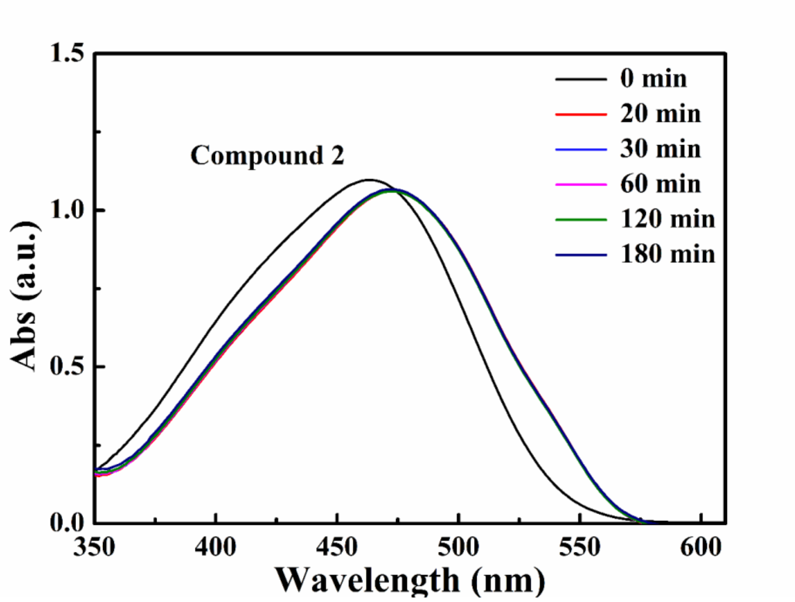

Fig. S42.Compound **2** was used to adsorb MO aqueous solution

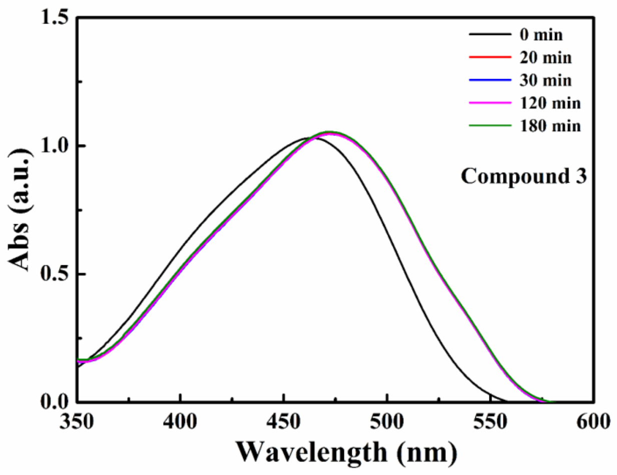

Fig. S43.Compound **3** was used to adsorb MO aqueous solution

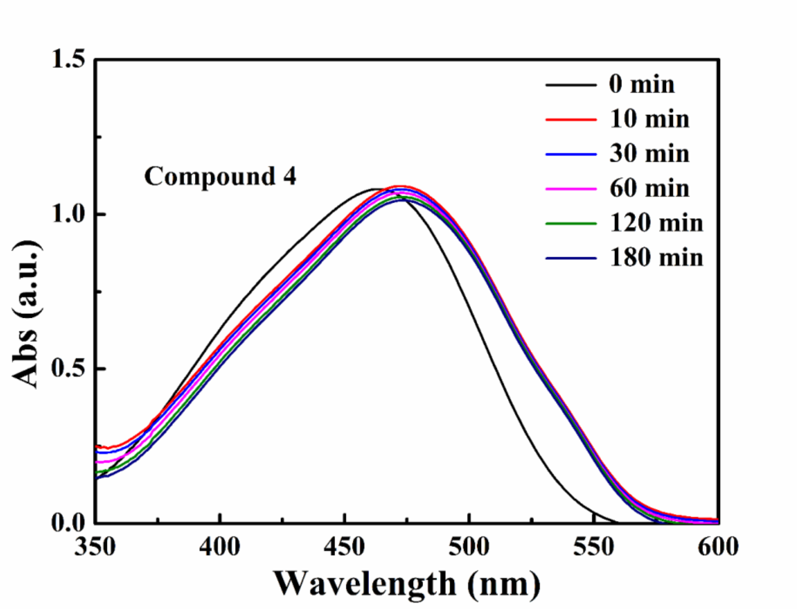

Fig. S44.Compound **4** was used to adsorb MO aqueous solution

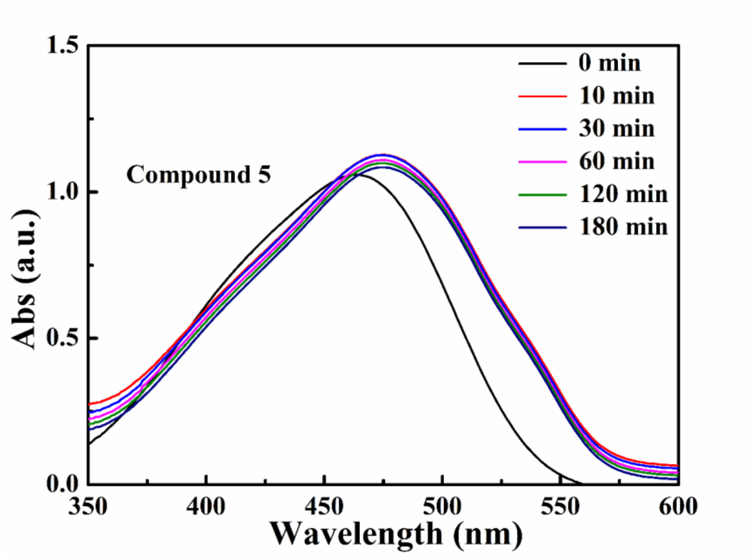

Fig. S45.Compound **5** was used to adsorb MO aqueous solution

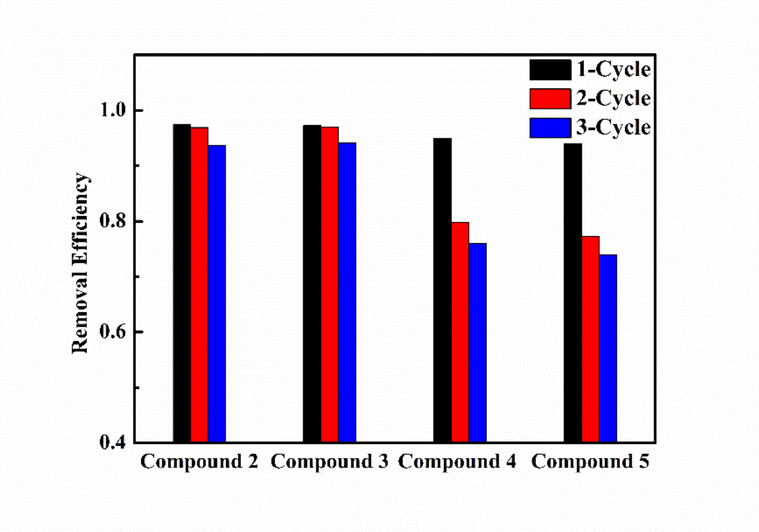

Fig. S46: Recycling tests of Compound 2-5 toward MB adsorption

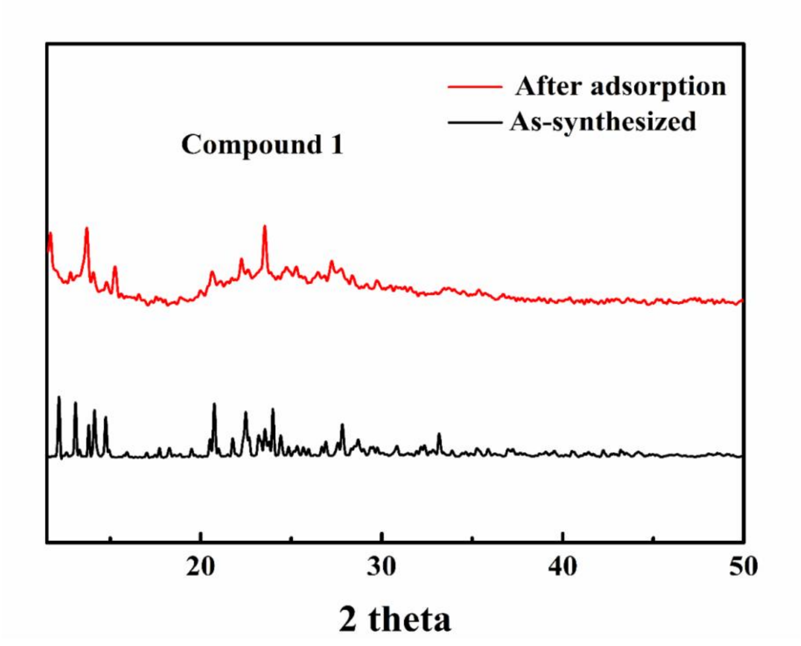

Fig. S47. Experimental and after adsorption XRD spectra of compound 1

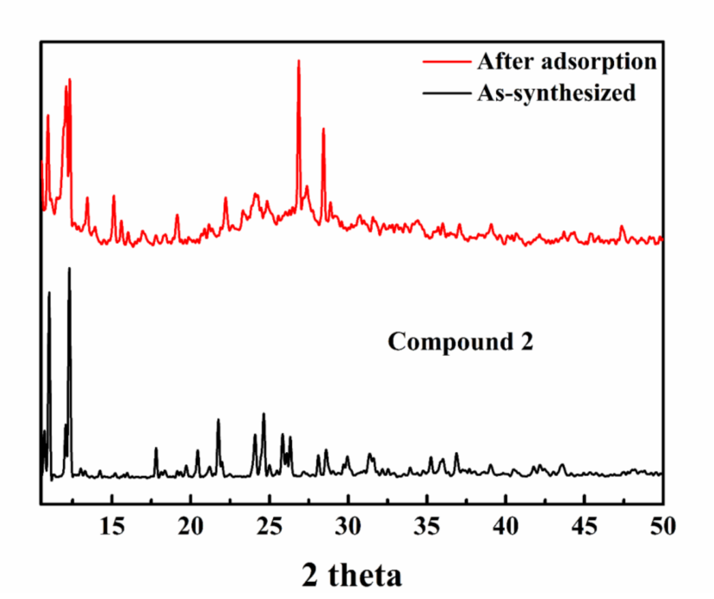

Fig. S48. Experimental and after adsorption XRD spectra of compound 2

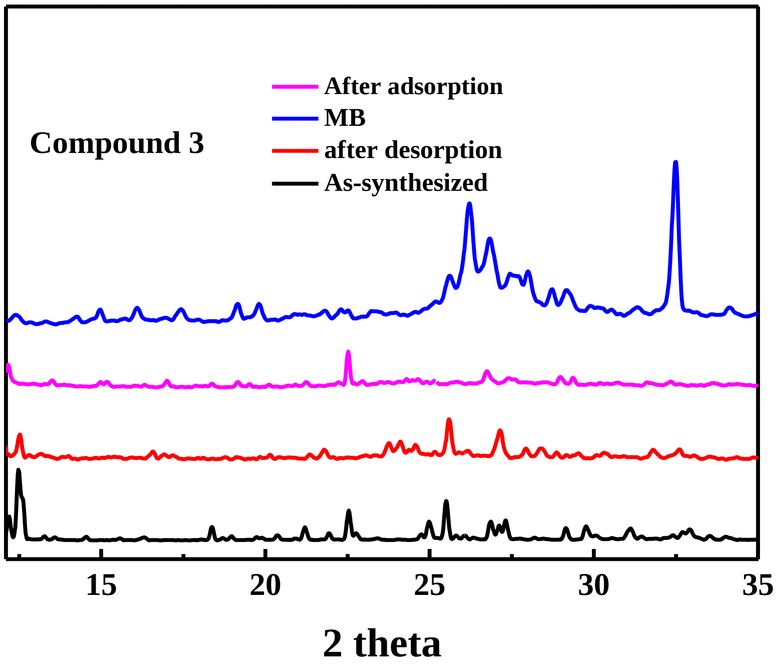

Fig. S49. Experimental and after adsorption XRD spectra of compound 3

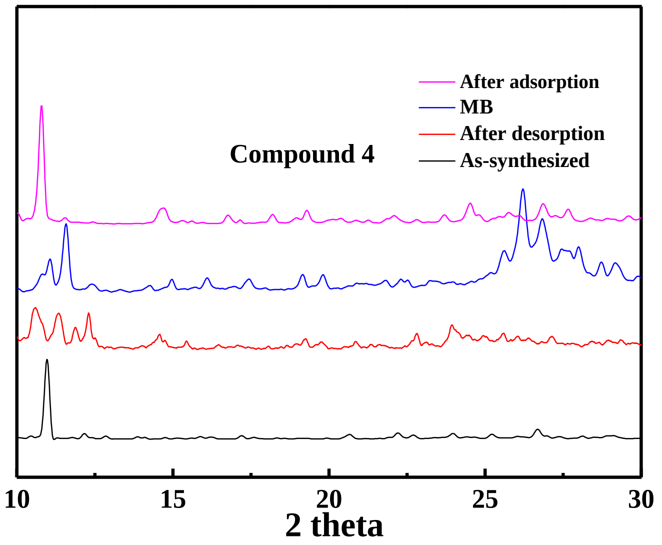

Fig. S50. Experimental and After adsorption XRD spectras of compound 4

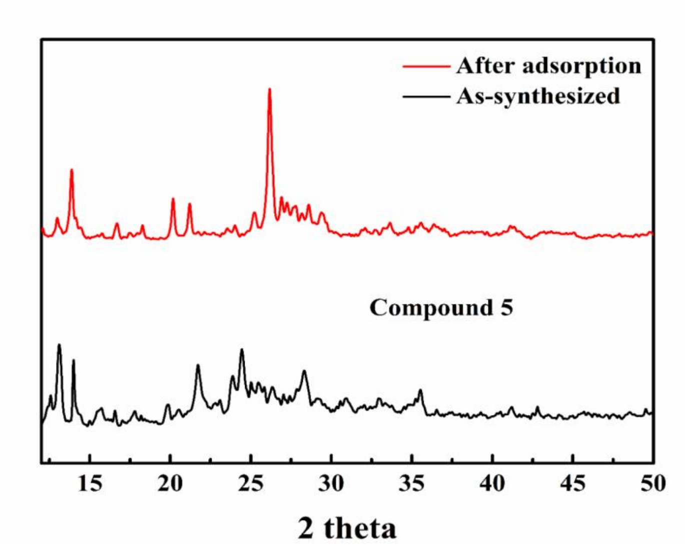

Fig. S51. Experimental and after adsorption XRD spectras of compound 5

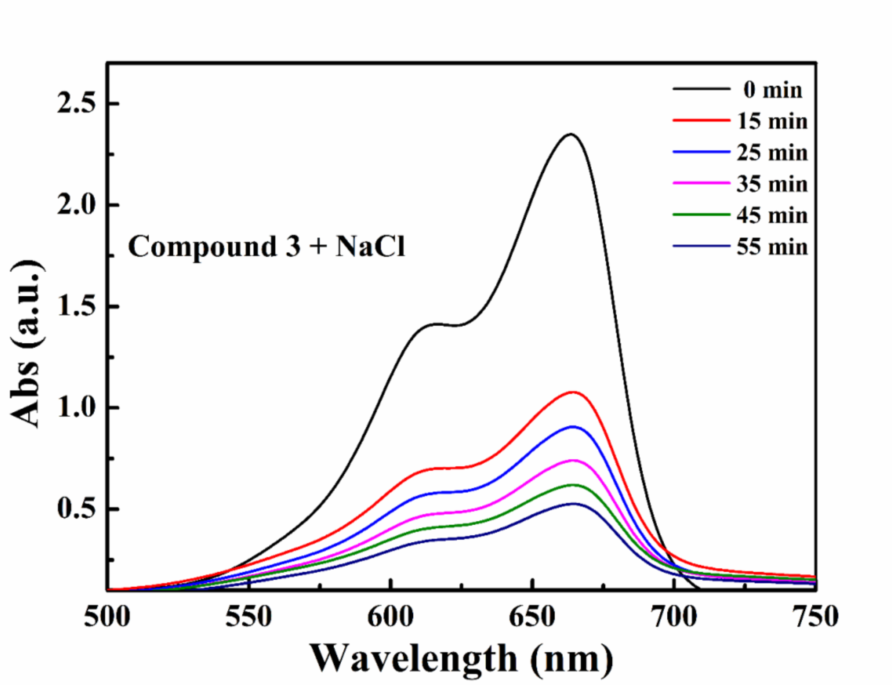

Fig. S52.Compound **3** was used to adsorb MB+NaCl aqueous solution
